# Genomic innovations in cancer prevention, diagnosis, prognosis and precision therapeutics

**DOI:** 10.3389/fgene.2026.1828450

**Published:** 2026-07-06

**Authors:** Hanumappa Ananda, Sadhu R. Sahana, Shruthi R. Murthy, Amrutha N. Kunnath, Akila Prashant, Jussuf T. Kaifi, Pura K. Kiran, Kanve N. Suvilesh

**Affiliations:** 1 Department of Surgery, University of Connecticut School of Medicine, Farmington, CT, United States; 2 Department of Medical Genetics, JSS Medical College and Hospital, JSS Academy of Higher Education and Research, Mysore, Karnataka, India; 3 Division of Reproductive Biology, Department of Reproductive Science, Kasturba Medical College, Manipal Academy of Higher Education, Manipal, India; 4 Department of Biochemistry, JSS Medical College and Hospital, JSS Academy of Higher Education and Research, Mysore, Karnataka, India; 5 Department of Surgery, Ellis Fischel Cancer Center, University of Missouri, Columbia, MO, United States; 6 Department of Medical Oncology, JSS Medical College and Hospital, JSS Academy of Higher Education and Research, Mysore, Karnataka, India

**Keywords:** cancer genomics, cancer vaccines, CAR-T therapy, clinical trials, companion diagnostics, CRISPR-based cancer therapy, immunogenomics, neoantigens

## Abstract

Cancer research has undergone a transformative change with the advent of high-throughput genomic technologies. Advances in next-generation sequencing accelerated the identification of somatic and germline alterations that drive tumorigenesis enabling the transition from traditional histology-based cancer classification to molecularly informed precision oncology. Large-scale sequencing initiatives and clinical genomic profiling facilitated the development of companion diagnostic assays and targeted therapies. Beyond targeted therapies, genomic innovations have also catalyzed the emergence of novel therapeutic strategies including immunogenomics-driven immunotherapies, RNA-based therapeutics, cancer vaccines and genome editing technologies based on CRISPR-Cas systems. This review summarizes the major technological developments in cancer genomics, including sequencing platforms, transcriptomic profiling, liquid biopsy, and functional genomic screening, and highlights the utility of these innovations in discovery of actionable biomarkers and next-generation therapeutic strategies. Collectively, these advances underscore the central role of genomic technologies in driving the evolution of precision oncology toward more personalized and effective cancer treatment strategies.

## 1 Introduction

**FIGURE 1:**
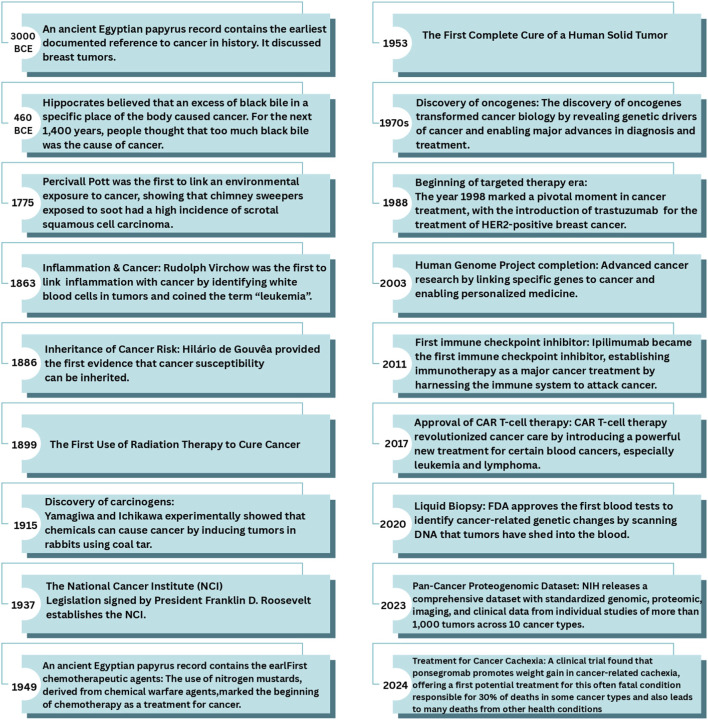
Historic developments and milestones in cancer. Timeline illustrates the major milestones in historical understanding of cancer, spanning from ancient observations to modern precision oncology. It traces the evolution of ideas from early descriptions of tumors in ancient Egypt and Hippocratic theories through the recognition of environmental and hereditary risk factors, development of radiation, chemo, targeted, and immunotherapies. Together, these milestones highlight how cancer research has progressed from descriptive pathology to molecular, genomic, and personalized medicine.

Advances in genome sequencing technologies have catalyzed a profound transformation in modern biomedical research and clinical oncology (Isaic et al., 2025). Historic developments and contributions by scientific communities incrementally laid the foundation for the integration of advanced techniques such as next-generation sequencing in cancer care (Figure 1). Over the past decade, the rapid decline in the cost of high-throughput next-generation sequencing has accelerated the global expansion of large-scale genomics initiatives, fundamentally reshaping how biological systems and diseases are studied and interpreted (Satam et al., 2023). What was once confined to research laboratories has now become an integral component of clinical workflows, particularly in the diagnosis and management of cancer, rare genetic disorders, and population-scale genomic studies.

In oncology, the integration of comprehensive genomic profiling has driven a paradigm shift from histopathology-based classification toward molecularly defined tumor subtypes (Das et al., 2026). Solid tumors are increasingly recognized as genomically heterogeneous entities characterized by complex patterns of somatic mutations, copy number alterations, structural variants, and gene fusions (Goldrich et al., 2021). Whole-genome sequencing, whole-exome sequencing, and targeted sequencing panels now enable systematic identification of oncogenic drivers, tumor suppressor alterations, and predictive biomarkers that inform prognosis and therapeutic decision-making (Zalis et al., 2024). Consequently, genomic profiling has become a cornerstone of precision oncology, guiding the selection of targeted therapies and shaping personalized treatment strategies.

Despite these advances, the exponential growth of genomic data presents substantial analytical and interpretive challenges (Olawade et al., 2025). Traditional bioinformatics pipelines and rule-based variant interpretation frameworks struggle to scale with the volume, complexity, and clinical urgency of tumor sequencing data (Zubair et al., 2025). Moreover, translating genomic alterations into clinically actionable insights requires integration of multidimensional datasets, robust annotation frameworks, and context-specific biological interpretation (Vitorino, 2024). These challenges are particularly pronounced in solid tumors where intratumoral heterogeneity and dynamic clonal evolution under therapeutic pressure further complicate data interpretation.

In this context, innovations in computational genomics, integrative bioinformatics, and data-driven decision-support systems are increasingly critical for realizing the full clinical potential of tumor genomic profiling (Jamalinia and Weiskirchen, 2025). When effectively leveraged, these approaches not only enable precise molecular characterization of solid tumors but also establish the foundation for the rational development and deployment of targeted genomic therapies. This review focuses on the technological and analytical advances underpinning genomic profiling of solid tumors and examines how these innovations have directly driven the evolution of targeted therapeutic strategies, resistance monitoring, and emerging precision oncology paradigms.

## 2 Next-generation sequencing: foundational technology driving modern genomics

Next-Generation Sequencing (NGS) evolved from the classical Sanger chain-termination method, which established the conceptual and technical foundation for DNA sequencing in the 1970s and enabled the first small-scale and later automated large-scale sequencing projects (Sanger et al., 1977). The first generation of NGS technologies appeared in the mid-2000s and transformed throughput and cost. A milestone was the demonstration of massive parallel pyrosequencing in picolitre reactors achieving orders-of-magnitude higher throughput relative to capillary electrophoresis and marked the acceleration of high-throughput sequencing (Margulies et al., 2005).

Shortly afterward, reversible-terminator chemistry implemented on dense flow cells (the solexa/Illumina approach) was scaled to produce accurate whole-human-genome data at dramatically reduced cost per base. This landmark work demonstrated accurate whole human genome sequencing using reversible terminator chemistry and established short-read sequencing as the dominant platform for whole genome sequencing (WGS) and many other applications. As technical capabilities advanced, NGS moved beyond short reads. Long-read, single-molecule technologies such as Pacific Biosciences and Oxford Nanopore enabled much longer contiguous reads, improved assembly of repetitive regions, native detection of base modifications, and better structural variant calling capabilities that address limitations of short-read sequencing and have steadily matured into clinically useful workflows (Ardui et al., 2018; Bowden et al., 2019). Hybrid linked-read, and long-read sequencing approaches improve haplotype phasing and structural variant resolution, while increased speed and portability are expanding the use of sequencing in point-of-care diagnostics, such as real-time nanopore-based applications in field and outbreak setting (Zheng et al., 2023; Gupta and Verma, 2019). Together these developments expand the range of clinically actionable variants, improve diagnostic yield in rare disease and cancer, and accelerate the discovery of novel therapeutic targets.

**FIGURE 2:**
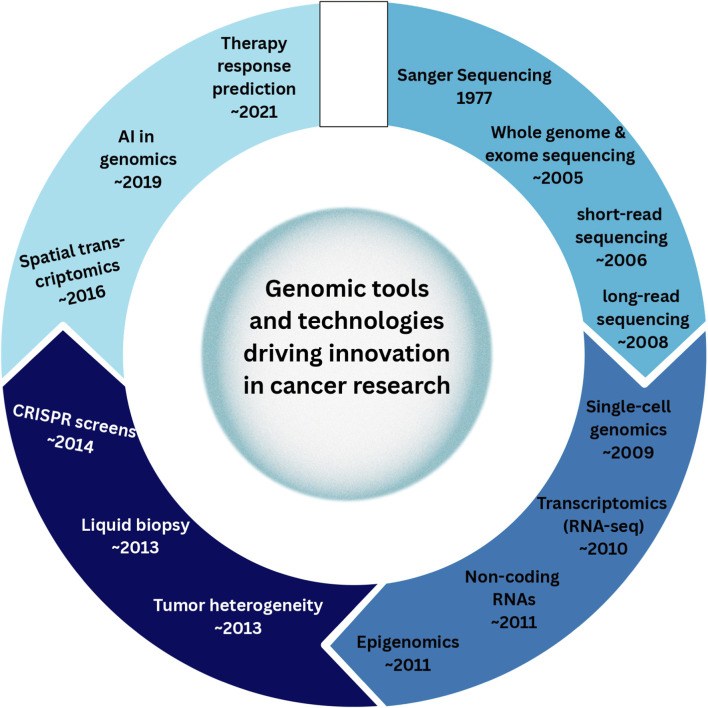
Genomic tools driving innovation in cancer research, diagnosis and treatment. The schematic timeline highlights key advances in sequencing, functional genomics, and computational approaches that have progressively transformed cancer research enabling the study of tumor heterogeneity, regulatory mechanisms, and precision therapy response prediction.

Comprehensive literature published around last 2 decades clearly articulates the technological trajectory from single-gene projects to genome-scale investigations and empowerment of new applications (e.g., transcriptomics, ChIP-seq, and metagenomics) (Mardis, 2008; Mardis, 2013). Besides being the foundation, NGS continues to advance genomic tools such as whole genome sequencing (WGS), whole exome sequencing (WES), RNA-sequencing, single cell DNA/RNA sequencing, spatial sequencing, liquid biopsy, epigenomics, and metagenomics (Table 1). Together, these tools drive innovation in cancer treatment (Figure 2).

**TABLE 1:** Comparative overview of genomic and precision oncology technologies in cancer research and clinical oncology.

Technology	Advantages	Limitations	Clinical utility	References
WES/WGS	High-throughput detection of SNVs, indels, CNVs, structural variants, and enables comprehensive genomic profiling	Expensive infrastructure, complex bioinformatics workflows, and data interpretation challenges	Comprehensive tumor profiling, actionable biomarker identification, and CDx development	Milbury et al. (2022), Allegretti et al. (2018)
RNA-seq	Detects fusion transcripts, alternative splicing events, gene-expression signatures, and non-coding RNAs	RNA instability in FFPE tissues, pre-analytical variability, and computational complexity	Fusion detection, prognostic stratification, transcriptomic profiling, and therapeutic target identification	Cieślik and Chinnaiyan (2018), Heyer et al. (2019), Siddaway et al. (2025), Capone et al. (2022)
scRNA-seq/ Spatial genomics	Resolves intratumoral heterogeneity and cellular interactions at single-cell resolution while preserving spatial architecture	High cost, limited scalability, and substantial computational burden	Characterization of TME, clonal evolution, immune profiling, and resistance mechanisms	Shi et al. (2025), Janesick et al. (2023), Shafighi et al. (2024)
Liquid biopsy	Minimally invasive; enables longitudinal and real-time monitoring of tumor evolution	Reduced sensitivity in low-shedding or early-stage tumors; potential false negatives	Minimal residual disease (MRD) detection, resistance monitoring, ctDNA profiling, and treatment-response assessment	Ma et al. (2024), Kaczmarek et al. (2025), Peng et al. (2021)
CRISPR and functional genomic screening	Enables systematic identification of cancer vulnerabilities, synthetic lethal interactions, and therapeutic targets	Potential off-target effects, delivery limitations, and ethical/regulatory concerns	Functional genomics, drug target discovery, resistance profiling, and genome engineering	Kim et al. (2024), He et al. (2026)
Companion diagnostics (CDx)	Facilitates biomarker-guided therapeutic selection and personalized treatment strategies	Requires rigorous analytical validation, regulatory approval, and standardized testing workflows	Precision treatment selection, patient stratification, and therapy-response prediction	Andrews et al. (2025)
Immunogenomics	Predicts immunotherapy response and characterizes tumor–immune interactions	Immune heterogeneity and variability in biomarker reproducibility across patients	PD-1/PD-L1 biomarker assessment, neoantigen discovery, and immunotherapy optimization	Liu and Mardis (2017), Garg et al. (2024)

## 3 Genomic tools and technologies driving innovation

### 3.1 Whole-genome and whole-exome sequencing: from concept to cornerstones of cancer genomics

WGS/WES has been central to the rise of precision genomic medicine and to transforming oncology from histology-driven to genotype-driven therapy. Whole-exome sequencing (WES) emerged as an efficient, cost-effective strategy to focus on the ∼1–2% of the genome that encodes proteins. The first successful application of exome capture and sequencing to identify the genetic cause of Miller syndrome, a Mendelian disorder, provided a proof-of-principle that WES could accelerate gene discovery for rare diseases (Ng et al., 2010).

Coordinated sequencing consortia harnessed WGS/WES to map disease-relevant somatic and germline variation at scale. The Cancer Genome Atlas (TCGA) and related projects applied WGS/WES and RNA-sequencing across thousands of tumors to catalogue recurrent somatic mutations, copy-number changes, and gene expression patterns, creating the genomic atlases that underpin modern cancer genomics (Tomczak et al., 2015). At population scale, projects such as the 1000 Genomes Project exploited high-throughput sequencing to map human genetic variation across populations and to provide critical reference data for variant interpretation in both research and clinical settings (1000 Genomes, Project Consortium, et al., 2010). These datasets, together with technological and analytical improvements, enabled robust clinical translation of gene panels and companion diagnostics. For example, approval of multi-gene NGS assays by the United States Food and Drug Administration (US-FDA) formalized the clinical utility of sequencing for diagnostic, prognostic, and therapy-selection uses (Milbury et al., 2022).

Early discoveries of recurrent, actionable driver alterations such as activating epidermal growth factor receptor (EGFR) kinase mutations in non-small-cell lung cancer that predict sensitivity to EGFR tyrosine-kinase inhibitors (Lynch et al., 2004), B-type rapidly accelerated fibrosarcoma (BRAF) V600E mutations in melanoma that enabled RAF/ mitogen-activated extracellular signal-regulated kinase (MEK) directed therapies (Davies et al., 2002), and anaplastic lymphoma kinase (ALK) fusion oncogenes in lung cancer that led to ALK inhibitors (Soda et al., 2007) illustrate how identifying specific genomic lesions directly produced new therapeutic strategies. More broadly, WES/WGS and deep targeted NGS panels have enabled: (i) robust biomarker discovery for targeted agents and immunotherapies, (ii) detection of resistance mechanisms such as secondary mutations and bypass pathways that guide sequential therapy, and (iii) patient selection for clinical trials and trial designs that match rare variants to drugs irrespective of tissue of origin. Comprehensive cancer sequencing atlases and routine US-FDA approved clinical NGS panels together established the infrastructure for molecular tumor boards and genomic guided therapeutic decision-making (Tomczak et al., 2015; Allegretti et al., 2018).

Overall, WES and WGS are widely accessible, drives population and disease genomics consortia such as 1000 Genomes and TCGA that directly enables the genomic identification of actionable cancer drivers leading to targeted therapies and precision oncology programs. The continuing integration of WGS/WES into clinical workflows promises further gains such as precise stratification of patients, earlier detection of actionable mutations and resistance, and a rapidly enlarging catalogue of genomic biomarkers that can be matched to increasingly specific targeted interventions.

### 3.2 RNA-sequencing in oncology: from transcriptomic landscapes to therapeutic actionability

#### 3.2.1 Coding RNAs

**FIGURE 3:**
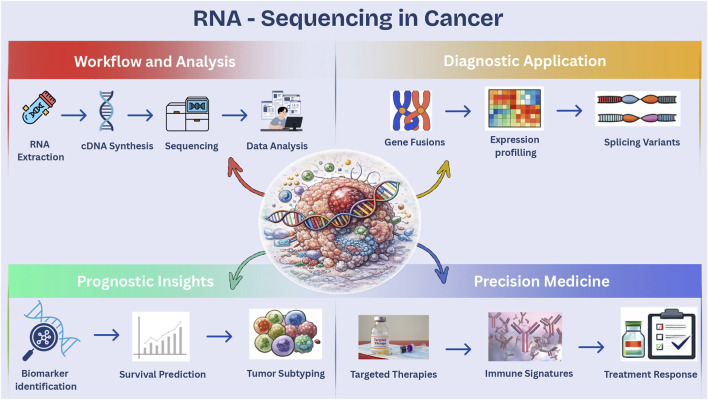
RNA-sequencing in cancer diagnosis, prognosis and precision medicine. Figure illustrates the workflow from RNA extraction to data analysis leading to diagnostic, prognostic and precision therapeutics insights.

RNA-sequencing (RNA-seq) is a high-throughput NGS methodology that profiles the whole transcriptome by sequencing complementary DNA (cDNA) generated from cellular RNA. This technology enables quantification of gene expression levels, detection of alternative splice variants, non-coding RNAs, allele-specific expression, and identification of chimeric or fusion transcripts across the cancer transcriptome (Figure 3). By capturing the dynamic RNA output of tumor cells, RNA-seq provides a functional readout of genomic activity that complements DNA sequencing and links genotype to phenotype. RNA-seq has largely replaced hybridization-based microarrays due to its greater sensitivity, dynamic range, and ability to detect novel transcripts (Cieślik and Chinnaiyan, 2018).

RNA-seq has demonstrated clinical value in cancer diagnosis by improving the detection of molecular alterations that are difficult to resolve with DNA-based assays alone. One of the earliest translational impacts of RNA-seq was the detection of oncogenic fusion genes, which are key diagnostic biomarkers in many hematologic malignancies and solid tumors. Traditional techniques such as fluorescence *in situ* hybridization, RT-PCR, or immunohistochemistry require prior knowledge of specific targets; in contrast, RNA-seq enables genome-wide discovery of known and novel fusion transcripts (Heyer et al., 2019). For example, integration of RNA-seq into clinical diagnostics has increased diagnostic yield compared with conventional methods, identifying fusion transcripts that refine or alter initial diagnoses and guide subsequent clinical decision-making (Figure 3). In prospective pan-cancer cohorts, targeted RNA-seq identified diagnostic alterations in a substantial fraction of cases, leading to modified therapeutic strategies based on molecular findings (Siddaway et al., 2025). Additionally, RNA-seq assists in tumor classification by transcriptomic subtyping. Distinct expression profiles can distinguish histological subtypes with clinical relevance, aiding in diagnosis and potentially informing therapy selection beyond single gene markers (Cieślik and Chinnaiyan, 2018).

One of the most important clinical applications of RNA-seq is the detection of gene fusions which often function as primary oncogenic drivers. Fusion events are especially prominent in leukemia, sarcomas, and subsets of solid tumors such as lung adenocarcinoma (e.g., EML4-ALK, KIF5B-RET) and prostate cancer (e.g., TMPRSS2-ERG) (Darabi et al., 2023). RNA-seq complements targeted DNA assays for fusion discovery because of the capability to sequence transcribed fusion junctions directly, allowing precise identification of both fusion genes and expression levels (Figure 3). RNA-seq has been shown to identify clinically relevant fusion transcripts not detected by traditional assays, thereby enhancing molecular diagnosis and expanding therapeutic options (Hehir-Kwa et al., 2022). In rare tumors such as myeloid sarcoma, RNA-seq-derived fusion profiles have been used for risk stratification, with recurrent fusion gene combinations correlating with poorer survival, highlighting the ability of RNA-seq in refining prognostic categorization and guiding the treatment (Yang et al., 2023). In routine diagnostic workflows, targeted RNA-seq panels have demonstrated high diagnostic yield in solid tumors, with a significant proportion of cases yielding actionable fusion events. An RNA-based NGS fusion assay identified fusion events in over 24% of 321 solid tumor cases, refining diagnoses in more than 30% of diagnostic specimens, and supporting its utility in broad histopathologic contexts (Fei et al., 2024).

Beyond structural changes detection, RNA-seq provides prognostic insights by capturing expression patterns associated with clinical outcomes (Figure 3). Gene expression signatures derived from RNA-seq can be used to stratify patients into prognostically distinct groups. Additionally, RNA-seq helps in identification of druggable targets that are involved in anti-cancer therapy resistance mechanisms (Suvilesh et al., 2024). RNA-seq is integral to precision oncology by identifying targetable alterations, expression of therapeutic targets, and resistance mechanisms. Transcriptome data can reveal overexpression of kinase drivers, alternative splicing events generating neoepitopes, or immune signatures predictive of immunotherapy response. These features inform choice of targeted agents, immune modulators, or combination regimens tailored to an individual’s tumor biology. Large-scale clinical experiences have demonstrated that RNA-seq yields clinically actionable data for a high proportion of patients. In a prospective pan-cancer cohort of >2,300 tumors, RNA-seq provided molecular data for 87% of patients including alterations that led to treatment changes with targeted therapies (Siddaway et al., 2025). While highly informative, RNA-seq is not without challenges. RNA stability, particularly in formalin-fixed paraffin-embedded (FFPE) samples, can affect data quality, and comprehensive bioinformatic pipelines are required to distinguish true fusion events from artifacts. Nonetheless, ongoing assay optimizations, development of new fixatives that can overcome the limitations of formalin fixation and computational improvements continue to enhance robustness and clinical utility (Capone et al., 2022; Jones et al., 2019; Bageritz et al., 2023; Yin et al., 2021; Li et al., 2018). Overall, RNA-sequencing has become an indispensable NGS tool in oncology that complements DNA-based profiling by providing functional insights into tumor biology. Through accurate gene expression quantification, fusion detection, prognostic gene expression signatures, and integration into precision medicine frameworks, RNA-seq enriches diagnostic, prognostic, and therapeutic decision-making in cancer care.

#### 3.2.2 Noncoding RNAs

Non-coding RNAs (ncRNAs) are RNA molecules that are transcribed from the genome but are not translated into proteins. They constitute the majority of the human transcriptome, with protein-coding genes representing ∼2% of the genome (Diamantopoulos et al., 2018). Numerous cellular functions, including development, differentiation, and disease, are influenced by ncRNAs. Advances in RNA-seq have revealed that ncRNAs are significant regulators of gene expression and play essential roles in development, differentiation, and disease (Micheel et al., 2021). There are several types of ncRNAs that are broadly grouped into 3 major classes as small ncRNAs (sncRNAs), long non-coding RNAs (lncRNAs), and circular RNAs (circRNAs), which all have a role in controlling the development and progression trajectories of malignancies (Slack and Chinnaiyan, 2019). sncRNAs include microRNAs (miRNAs), small interfering RNAs (siRNAs), and PIWI-interacting RNAs (piRNAs) and are less than 200 nucleotides in size (Klimenko, 2017). lncRNAs are transcripts that are longer than 200 nucleotides with regulatory functions at transcriptional and post-transcriptional levels (Kesheh et al., 2022). circRNAs are covalently closed RNA molecules generated by back-splicing events, often functioning as miRNA sponges or transcriptional regulators (Garraffo and Beltran, 2025). Recent studies have shown that ncRNAs can be both prognostic and diagnostic indicators for cancer (Uppaluri et al., 2023).

RNA-seq enables comprehensive detection and quantitative profiling of ncRNAs, through high-throughput generation of millions of sequences that are computationally aligned to a reference genome (Uchida, 2017; Grillone et al., 2020). A total RNA-seq approach through ribosomal RNA-depletion rather than poly-A selection is required for the comprehensive profiling as many ncRNAs lack poly-A tails. Additionally, to capture small RNAs like miRNA, specialized library preparation focusing on size selection is needed (Tripathi et al., 2017; Micheel et al., 2021). miRNAs are ∼18–25 nucleotide RNAs that regulate gene expression through post-transcriptional repression by binding to complementary sequences in target mRNAs (O’Brien et al., 2018). Identification of deletion of miR-15a and miR-16-1 in chronic lymphocytic leukemia established the first direct link between miRNAs and cancer (Calin et al., 2002; Cimmino et al., 2005). This discovery marked a pivotal milestone demonstrating that miRNAs can function as tumor suppressors. Further studies demonstrated that miRNAs could act either as onco-miRNAs by promoting tumorigenesis or as tumor suppressor miRNAs by inhibiting oncogenic pathways (Svoronos et al., 2016). miRNA expression signatures can discriminate tumor types, subtypes, and tissue of origin with high specificity. Circulating miRNAs detected in plasma or serum are stable and resistant to RNase degradation, making them attractive minimally invasive biomarkers (Søkilde et al., 2014; Singh et al., 2024; Visone and Croce, 2009). Their utility has been demonstrated in early cancer detection and monitoring disease recurrence (Chacko and Ankri, 2024; Macha et al., 2014; Jelski and Mroczko, 2023). Distinct miRNA expression profiles correlate with patient survival, metastatic potential, and therapeutic response across multiple cancers. For example, overexpression of miR-21 has been associated with poor prognosis in several solid tumors (Selcuklu et al., 2009; Huang et al., 2015). Some ncRNAs are highly expressed in saliva, serum, urine and stool of cancer patients and can serve as diagnostic markers or prognostic indicators (Toden and Goel, 2022; Xie et al., 2016; Zhan et al., 2018; Gharib et al., 2021; Zhou et al., 2015).

lncRNAs regulate gene expression through chromatin remodeling, transcriptional modulation, RNA sponging, and protein scaffolding functions (Zhang et al., 2019). One of the earliest and most studied oncogenic lncRNAs, hox transcript antisense intergenic RNA (HOTAIR), was shown to reprogram chromatin states and promote metastasis in breast cancer, representing a major milestone in understanding lncRNA-mediated tumor progression. Further studies clearly demonstrated that elevated HOTAIR expression correlates with metastasis and poor survival in multiple cancers (Nazari et al., 2024; Chen L. et al., 2021; Toy et al., 2019). Similarly, other lncRNAs have been incorporated into prognostic risk models using transcriptomic datasets (Pinkney et al., 2024; Xu et al., 2021; Zhao et al., 2020). lncRNAs are emerging as predictive biomarkers of therapeutic response. Their involvement in drug resistance pathways, including modulation of epithelial-mesenchymal transition (EMT) and immune signaling, positions them as potential targets for combination therapy strategies (Peña-Flores et al., 2022; Zhang H. et al., 2025; Zhang D. et al., 2025; Lampropoulou et al., 2023). Targeted silencing using antisense oligonucleotides and CRISPR-based approaches represents a developing frontier in lncRNAs based therapeutics.

circRNAs are generated through a special type of alternative splicing termed back-splicing and are highly stable due to their covalently closed structure (Saaoud et al., 2021). Many circRNAs function as miRNA sponges thereby indirectly regulating oncogenic signaling pathways (Hou and Zhang, 2017). Their abundance and stability in body fluids make them promising non-invasive biomarkers for cancer detection and monitoring (Patop et al., 2019). Recent studies suggest that circRNAs may also encode functional peptides or regulate transcription directly, expanding the conceptual framework of ncRNA biology in cancer (Xu et al., 2025). Principally, ncRNAs have emerged as fundamental regulators of cancer biology with significant clinical implications. From early diagnostic applications and prognostic stratification to the development of RNA-based therapeutics, ncRNAs are now a critical component of precision oncology. Continued integration of transcriptomic profiling, functional genomics, and RNA-targeted drug development will further refine the role of ncRNAs in personalized cancer management.

### 3.3 Single-cell and spatial genomics: decoding tumor architecture

**FIGURE 4:**
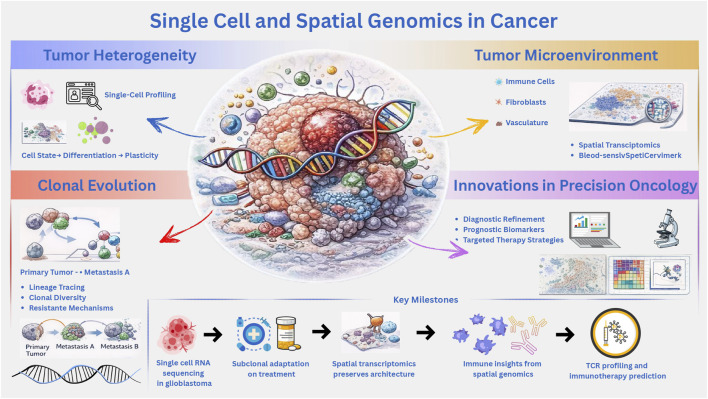
Single cell and spatial genomics as advanced tools in cancer. Importance of single cell and spatial genomics in studying tumor heterogeneity, microenvironment, and clonal evolution that collectively resulted in key milestones achievement in oncology.

Single-cell and spatial genomics technologies represent transformative advances in NGS (Figure 4). They enable high-resolution dissection of the cellular and microenvironmental complexity of tumors (Molla Desta and Birhanu, 2025). Bulk RNA-seq provides average transcriptomic changes across mixed cell populations whereas single-cell sequencing offers information about genomic/transcriptomic changes of individual cell type and states (Li and Wang, 2021). Spatial genomics preserves tissue architecture and locational framework of cell type interactions within the tumor microenvironment (TME) (Molla Desta and Birhanu, 2025; Piwecka et al., 2023). Together, single-cell and spatial genomic technologies have opened new frontiers in cancer biology by revealing tumor heterogeneity, delineating clonal evolution trajectories, disclosing tumor-immune interactions, and enhancing diagnostic and prognostic precision (Shi et al., 2025). Additionally, recent advancements in spatial and single-cell omics have dramatically revolutionized biomarker identification in cancer immunotherapy by tackling fundamental issues such as immune evasion and variability within the inter and intra tumors (Molla Desta and Birhanu, 2025; Piwecka et al., 2023). Despite their relatively high costs as compared to other sequencing methods, they are increasingly used in oncology because of their capacity to detect heterogeneity among individual cells, discriminate between small numbers of cells, and delineate cell maps.

Single-cell RNA sequencing (scRNA-seq) and single-nuclear RNA sequencing (snRNA-seq) captures transcriptomes of individual cells or nuclei thereby enabling analysis of gene expression at single-cell or nuclei resolution (Suvilesh et al., 2022; Cuevas-Diaz et al., 2022). This approach typically involves tissue dissociation, single cell/nuclei isolation, cDNA library preparation, and sequencing. Computational pipelines then cluster cells into distinct populations based on expression profiles, identifying cell types and subtypes that drive tumor progression or therapy resistance (Suvilesh et al., 2022; Cuevas-Diaz et al., 2022). On the other hand, spatial transcriptomics integrate high-resolution sequencing with tissue spatial information, mapping gene expression back onto histological sections. Methods such as 10x Genomics Visium, Slide-Seq, and MERFISH® retain spatial context, bridging molecular profiles with histopathology (Janesick et al., 2023; Rodriques et al., 2019; Marshall et al., 2022; Xia et al., 2019). These technologies allow examination of tumor architecture, stromal niches, immune cell infiltration patterns, and spatial gene expression. Additionally, spatial transcriptomics provide ideas about crosstalk between tumor-immune cells, tumor-stromal cells that in turn shed light on dynamics of tumor changes.

Tumor heterogeneity arises from genetic diversity within cancer cell populations and from variability in stromal and immune components. Bulk profiling averages these changes, obscuring sub clonal and microenvironmental complexity. Single-cell genomics reveal distinct cellular subpopulations within tumors that correlate with varied proliferative capacities, differentiation states, and therapeutic sensitivities (Figure 4). Spatial genomics further dissects *in situ* organization and interaction of these subpopulations with surrounding cells. For example, scRNA-seq in glioblastoma has revealed cellular hierarchies with stem-like and differentiated subclones, each exhibiting distinct transcriptional programs (Neftel et al., 2019). Similarly, single-cell profiling in melanoma identified treatment-induced subpopulations associated with early adaptive resistance (Su et al., 2017). Other than tumor heterogeneity, single-cell and spatial technologies aid in studying clonal evolution trajectories by combining transcriptomic signatures with genetic mutations (Shafighi et al., 2024). Combined single-cell DNA and RNA sequencing allow identification of mutational events that drive clonal expansion and therapy resistance (Zhang W. et al., 2025). Analyses of acute myeloid leukemia demonstrated that pre-existing minor clones can expand post-therapy thereby contributing to relapse (Madaci et al., 2021). If the study included spatial genomics, it could have revealed specific clones occupying distinct niches within the TME that influence metastatic potential and treatment response. TME consists of immune cells, fibroblasts, endothelial cells, and extracellular matrix elements that interact dynamically with cancer cells. Single-cell and spatial approaches have redefined our understanding of the, identifying immunosuppressive cell states, exhausted T-cell phenotypes, and unique myeloid subpopulations that influence prognosis and therapeutic outcomes (Shi et al., 2025; de Visser and Joyce, 2023; Zhu et al., 2025; Gonzalez Castro et al., 2026). Spatial transcriptomics has the potential to reveal immune-stromal components such as tertiary lymphoid structures associated with favorable immunotherapy responses, and peritumoral niches enriched in suppressive immune cell types that correlate with poor outcomes (Deng et al., 2025).

Apart from their utility in studying tumor heterogeneity, clonal evolution and TME, single-cell and spatial genomics are highly useful in achieving diagnostic precision, prognostic patient stratification and precision medicine (Liang et al., 2024; Nath and Bild, 2021; Le et al., 2025). Single-cell and spatial profiling improve diagnostics by resolving tumor subtypes with distinct cellular compositions that may not be evident with bulk RNA-seq (Figure 4). For example, scRNA-seq can been used to classify subtypes of breast cancer enriched for basal-like versus luminal-like tumors (Ren et al., 2021). Spatial transcriptomics can identify histologically cryptic regions of tumor invasion and early dissemination in colorectal cancer improving detection sensitivity over conventional histopathology (Wood et al., 2023). Prognostically, single-cell gene expression signatures correlate with clinical outcomes. In triple-negative breast cancer, high proportions of immunosuppressive macrophages and exhausted T cells identified by scRNA-seq predicted poor survival and chemoresistance (Zhao et al., 2024; Xiao et al., 2025). Spatial profiling has further linked immune niches in three-dimensional space with prognosis in melanoma and lung cancer where localized T-cell enrichment predicts survival (Eliason et al., 2025; Sandström Gerdtsson et al., 2023). Single-cell and spatial genomics can also aid in precision medicine and therapeutic decision making by identifying therapeutic vulnerabilities and predicting response to therapy. T-cell receptor clonality and activation state discovered by scRNA-seq predicted durable response in lung cancer patients treated with checkpoint inhibitors (Shorer et al., 2025). Moreover, single-cell profiling has guided therapy design by identifying co-occurring signaling states and compensatory pathways that confer resistance to targeted agents enabling rational selection of kinase inhibitors (Ianevski et al., 2024).

Emerging and future directions of single-cell and spatial omics lie in multi-omic single-cell integration, spatially resolved profiling of TME combined with DNA and proteomic profiling and real-time monitoring and longitudinal profiling of tumor or seeds of tumor. Efforts to simultaneously profile RNA, epigenome, and protein at single-cell resolution are advancing. For example, cellular indexing of transcriptomes and epitopes by sequencing (CITE-seq) is a single-cell multi-omics technique that simultaneously profiles RNA and surface protein levels in individual cells (Song et al., 2025). Also, assay for transposase-accessible chromatin using sequencing (ATAC-seq) combined with scRNA-seq maps chromatin accessibility and gene expression simultaneously in individual cells to link epigenetics directly to functional gene output (Tang et al., 2025). Emerging platforms that combine spatial transcriptomics with protein or chromatin accessibility profiling have the potential to uncover TME states and cellular interactions in depth. Techniques such as spatial-ATAC-seq and multiplexed immunofluorescence integration with spatial transcriptomics are rapidly gaining traction. Additionally, longitudinal single-cell profiling from serial biopsies or circulating tumor cells can gain insight into clonal changes over time that in turn can help in early detection of metastasis and treatment response (Radfar et al., 2022; Zhang S. et al., 2021). Briefly, single-cell and spatial genomics have redefined the analysis of cancer biology by delivering unprecedented resolution of tumor cellular heterogeneity, clonal evolution, and microenvironmental interactions. By integrating these approaches into diagnostic and prognostic frameworks and by guiding precision therapeutic strategies, they have the potential to transform cancer research and clinical care. Continued technological innovation, particularly multi-omic and spatially resolved assays will further enhance our understanding of TME and improve patient-specific treatment paradigms.

### 3.4 Epigenomics: mapping gene regulatory alterations for therapeutic opportunities

**FIGURE 5:**
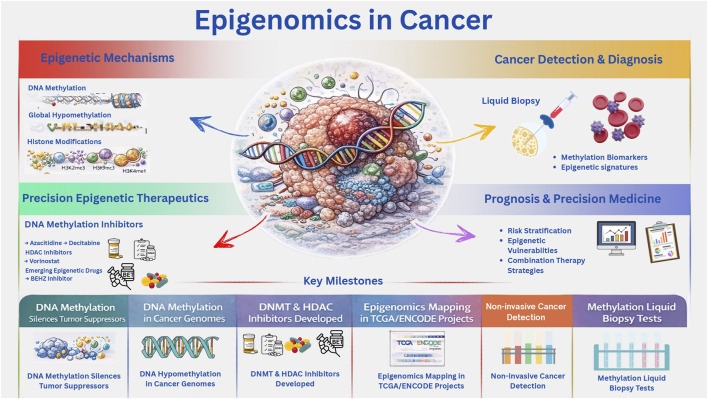
Epigenomic landscape in cancer. Role of epigenomics in cancer diagnosis, prognosis and precision therapeutics that resulted in key milestones achievement in cancer research and translational medicine.

Epigenetics refers to heritable and reversible modifications in gene expression that occur without alterations in the DNA sequence (Figure 5). These modifications include DNA methylation, histone modifications, chromatin remodeling, and regulation by non-coding RNAs (Al Aboud et al., 2026). Collectively, genome-wide mapping of these regulatory changes constitutes epigenomics and provides a comprehensive view of chromatin states and gene regulatory networks in cells. Epigenetic regulation plays a critical role in maintaining cellular identity and controlling gene expression during development (Skinner, 2011). Dysregulation of epigenetic mechanisms has been recognized as a hallmark of cancer contributing to tumor initiation, progression, metastasis, and therapeutic resistance (Fritz et al., 2022; Shenoy, 2025; Guo et al., 2022; Marei, 2025; Avella et al., 2022). Unlike genetic mutations, epigenetic alterations are potentially reversible which makes them attractive targets for therapeutic intervention (Kelly et al., 2010). Advances in NGS technologies have enabled high resolution mapping of epigenetic modifications across the genome through techniques such as whole-genome/exome bisulfite sequencing, chromatin immunoprecipitation sequencing (ChIP-seq), ATAC-seq, and methylation arrays (Li, 2021; O’Geen et al., 2011; Cooper et al., 2022; Volpe and Das, 2023) These technologies have significantly expanded our understanding of the epigenomic landscape of cancer.

DNA methylation is the most extensively studied epigenetic modification and involves the addition of a methyl group to the 5-carbon position of cytosine residues in CpG dinucleotides. Aberrant DNA methylation patterns are a defining feature of cancer genomes typically characterized by global hypomethylation and focal hypermethylation of promoter CpG islands (Paska and Hudler, 2015). Promoter hypermethylation can lead to transcriptional silencing of tumor suppressor genes contributing to tumorigenesis. Early studies demonstrated hypermethylation-mediated silencing of genes such as cyclin-dependent kinase inhibitor 2A (CDKN2A), MutL homolog 1 (MLH1), and breast cancer gene 1 (BRCA1) highlighting the role of epigenetic inactivation in cancer development (Myöhänen et al., 1998; Esteller et al., 1999; Catteau and Morris, 2002). Conversely, genome wide hypomethylation may promote chromosomal instability and activation of oncogenes further contributing to malignant transformation (Sheaffer et al., 2016). Similar to promoter methylation, histone proteins undergo modification that changes chromatin states. Histone proteins undergo multiple post-translational modifications including acetylation, methylation, phosphorylation, and ubiquitination (Liu et al., 2023). These modifications regulate chromatin structure and accessibility and thereby influence transcriptional activity. Dysregulation of histone-modifying enzymes such as histone methyltransferases and histone deacetylases (HDACs) have been implicated in cancer progression. For instance, mutations in epigenetic regulators such as enhancer of zeste homolog 2 (EZH2), lysine methyltransferase 2D (KMT2D), and AT-rich interaction domain 1A (ARID1A) have been identified across multiple malignancies emphasizing the importance of chromatin remodeling in oncogenesis (Verma et al., 2025; Kammarambath et al., 2025; Kearns et al., 2025). Chromatin accessibility profiling using ATAC-seq allows identification of regulatory elements such as promoters, enhancers, and super-enhancers that control gene expression. Alterations in any of these can drive oncogenic transcriptional programs and influence tumorigenesis.

Epigenomics is a very useful NGS based tool for early cancer detection, diagnosis, prognosis and precision therapeutics (Figure 5). Epigenomic biomarkers have emerged as powerful tools for early cancer detection due to their stability and tissue specificity. DNA methylation signatures are particularly promising diagnostic markers because they can be detected in circulating tumor DNA through minimally invasive liquid biopsies (Li and Sun, 2024). For example, methylation patterns of genes such as septin 9 have been successfully utilized for non-invasive colorectal cancer screening (Li Y. et al., 2023). Epigenetic alterations often occur at early stages of tumorigenesis preceding genetic mutations. This makes them valuable biomarkers for early cancer detection and risk assessment. Genome-wide methylation screening in circulating DNA has been explored for early detection. Furthermore, factors such as diet, smoking, and exposure to carcinogens can influence epigenetic states linking epigenomics with cancer prevention strategies. Epigenetic alterations can also provide valuable prognostic information (Figure 5). DNA methylation patterns often correlate with disease progression, metastatic potential, and patient survival. For instance, the CpG island methylator phenotype in colorectal cancer has been associated with distinct clinical outcomes and molecular features (Bae et al., 2017). Similarly, methylation-based prognostic signatures have been developed to stratify patients into risk categories in several malignancies including glioma, breast cancer, and lung cancer (Yang et al., 2022; Frontiers Production Office, 2020; Li et al., 2024). Genome-wide epigenomic profiling therefore provides insights into tumor aggressiveness and disease trajectory. Reversibility of epigenetic alterations is one of the most significant advantages that makes them attractive therapeutic targets (Figure 5). Several epigenetic drugs have been developed to modulate aberrant chromatin states in cancer. DNA methyltransferase inhibitors such as azacitidine and decitabine have been approved for treatment of myelodysplastic syndromes and certain leukemias (Xie et al., 2015; Wenger et al., 2026). Similarly, histone deacetylase inhibitors including vorinostat and romidepsin are used in the treatment of hematologic malignancies (Tiffon et al., 2011). Emerging therapeutic strategies include inhibitors targeting bromodomain proteins (BET inhibitors), histone methyltransferases, and chromatin remodeling complexes (Sarnik et al., 2021). Integration of epigenomic data with genomic and transcriptomic profiles enables identification of patient-specific epigenetic vulnerabilities and thereby supports precision oncology approaches.

### 3.5 Liquid biopsy: circulating biomarkers for precision oncology

**FIGURE 6:**
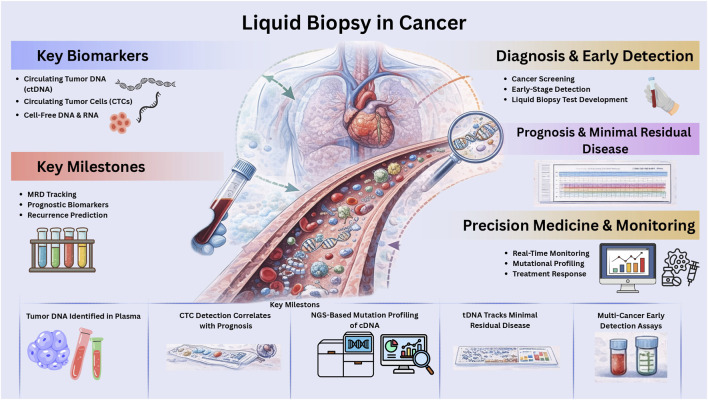
Liquid biopsy in cancer. Liquid biopsy in cancer diagnosis, prognosis and precision therapeutics paving the way for recent milestones in circulating biomarkers based bench to bedside transition.

Liquid biopsy refers to the analysis of tumor-derived biomarkers present in body fluids, most commonly blood, offering a minimally invasive alternative to conventional tissue biopsies (Connal et al., 2023). These biomarkers include circulating tumor DNA (ctDNA), circulating tumor cells (CTCs), cell-free DNA (cfDNA), cell-free RNA (cfRNA), extracellular vesicles such as exosomes, and tumor-derived proteins (Figure 6) (Ma et al., 2024). Unlike tissue biopsies which capture a spatially and temporally limited snapshot of a tumor, liquid biopsies can reflect the dynamic and systemic nature of tumor evolution by sampling tumor-derived biomarkers released from primary and metastatic lesions into circulation (Rouvinov et al., 2026). Advances in NGS have enabled highly sensitive identification of blood-based biomarkers thereby expanding the clinical utility of liquid biopsy in cancer detection, monitoring, and therapeutic decision-making (Figure 6). Accumulating evidence clearly suggests that sequencing blood-based biomarkers can be a powerful tool for real-time tumor profiling and longitudinal monitoring of disease progression (Ma et al., 2024; Kaczmarek et al., 2025; Lin et al., 2021).

cfDNA consists of fragmented DNA molecules released into circulation from apoptotic and necrotic cells. A fraction of cfDNA derived from tumor cells is referred to as ctDNA which carries tumor-specific genetic and epigenetic alterations such as point mutations, copy number variations, and methylation patterns (Dao et al., 2023). ctDNA analysis enables detection of tumor-specific mutations and provides insights into tumor burden, clonal evolution, minimal residual disease (MRD) and therapeutic resistance (Peng et al., 2021). High-throughput sequencing approaches allow comprehensive profiling of ctDNA to identify actionable mutations and guide targeted therapy selection (Caputo et al., 2022). CTCs are intact cancer cells shed from primary or metastatic tumors into the bloodstream (Lawrence et al., 2023). Detection and characterization of CTCs provide valuable insights into metastatic dissemination and tumor biology (Alix-Panabières and Pantel, 2025; Suvilesh et al., 2023). Enumeration of CTCs has been shown to correlate with prognosis in several malignancies, including breast, lung, prostate, and colorectal cancers (Zhang et al., 2012; Manjunath et al., 2022; Manjunath et al., 2019; Kuske et al., 2016; Hamid et al., 2021). Advances in single-cell sequencing technologies allow molecular profiling of CTCs enabling the study of tumor heterogeneity and metastatic potential at single-cell resolution (Suvilesh et al., 2022; Radfar et al., 2022). cfRNA including messenger RNA and non-coding RNAs such as miRNAs represent another important component of liquid biopsy (Kolenda et al., 2020). Tumor-derived RNAs circulate in plasma and can serve as biomarkers for cancer detection and monitoring. miRNAs are stable in circulation due to their association with protein complexes or extracellular vesicles which make them promising diagnostic and prognostic biomarker (Zhong et al., 2024). Extracellular vesicles (EVs) including exosomes are lipid bilayer-enclosed particles secreted by cells that carry nucleic acids, proteins, and lipids reflective of their cell of origin (Doyle and Wang, 2019). Tumor-derived exosomes play a critical role in intercellular communication and can modulate the tumor microenvironment, promote metastasis, and influence immune responses (Maheshwari et al., 2014). Exosomal nucleic acids and proteins can be isolated from blood and analyzed to identify tumor-specific molecular signatures that serve as a source of biomarkers for cancer diagnosis and monitoring (Paramanantham et al., 2025).

One of the most promising applications of liquid biopsy is early cancer detection through analysis of tumor-derived nucleic acids in blood. ctDNA-based assays can detect cancer-associated mutations and methylation signatures in screening patients enabling earlier diagnosis compared with conventional imaging or tissue biopsy methods (Chen K. et al., 2021). Methylation profiling of cfDNA has shown promise for early detection with the ability to identify tumor tissue of origin based on epigenetic signatures (Huang and Wang, 2019). Such approaches represent a major advancement toward population-level cancer screening using minimally invasive tests. Liquid biopsy based NGS testing also enables detection of MRD following surgery or therapy providing early indications of relapse before clinical or radiologic evidence becomes apparent (Honoré et al., 2021). In addition, liquid biopsy biomarkers provide important prognostic information across several cancer types. Elevated levels of ctDNA and CTCs have been associated with increased tumor burden, metastatic progression, and reduced overall survival in multiple cancer types. For example, persistent ctDNA following curative-intent surgery has been shown to predict recurrence in colorectal cancer months before radiographic detection highlighting its value as a prognostic marker (Ji et al., 2024). Liquid biopsy has become an integral component of precision oncology by enabling real-time monitoring of tumor genomics. ctDNA sequencing can identify actionable mutations, track emergence of resistance mutations, and guide therapeutic decision-making. For example, ctDNA-based detection of EGFR T790M mutations in non-small cell lung cancer allows identification of patients who may benefit from second or third-generation EGFR inhibitors (Bencze et al., 2022). In addition, liquid biopsy facilitates longitudinal sampling allowing clinicians to monitor clonal evolution and adapt treatment strategies accordingly. Integration of genomic, epigenomic, transcriptomic, and proteomic information from circulating biomarkers is emerging as a powerful strategy to enhance diagnostic accuracy and biological insight. Multi-analyte assays combining ctDNA mutations, methylation signatures, and protein biomarkers have potential to improve sensitivity for early cancer detection. Further, DNA/RNA sequencing of CTCs provides an unprecedented opportunity to investigate metastatic dissemination and treatment resistance mechanisms at cellular resolution.

### 3.6 CRISPR and functional genomic screens: uncovering cancer vulnerabilities

Functional genome wide screening is a powerful tool for understanding the genetic foundations of biological pathways. These screens employ genetics approach in which cellular phenotypes arising from genome-wide perturbations are analyzed (Li K. et al., 2023). Over the past decade new methodological discoveries have greatly advanced screening technologies. In particular, the advent of clustered regularly interspaced palindromic repeats (CRISPR) has enabled researchers to silence or overexpress genes in a robust and highly specific manner (Kim et al., 2024). Loss-of-function screens play an important role in drug discovery through which new drugs are developed to treat illnesses. In the target-based drug discovery approach a target gene that is correlated with a disease is first identified and chemical compounds are then screened for a desired therapeutic effect (He et al., 2026). Large scale loss-of-function screens often perturb large sets of genes to discover targets in an unbiased manner (Lord et al., 2020). For instance, gene disruptions in healthy cells that recapitulate a disease phenotype implicate the gene’s association with the disease. Alternatively, genetic disruptions in diseased cells (e.g., cancer) that cause a normal phenotype can mimic the therapeutic effect of a drug (Cetin et al., 2024). In addition to discovering novel drug targets, loss-of-function screens can also be used to improve existing therapies. Genes identified in the primary screen undergo a rigorous process of target validation to confidently determine whether the identified gene is directly linked to the phenotypic effect. Reproducing data in biologically relevant cell types, 3D cultures, iPS cells, or patient primary cells are needed to find significant correlations of a gene in disease. Another strategy is to create distinct guide RNA (gRNA) sequences for the same gene and observe the changes in phenotype (Lu et al., 2017). For example, CRISPR can be employed in a secondary screen if RNA interference was used in a primary screen or vice-versa.

The term synthetic lethality describes the genetic interaction between two genes wherein the removal of one gene alone is not fatal but, the simultaneous loss or suppression of both causes cell death. Synthetic lethality has grown to be a key component of precision oncology by providing a method to target cancer cells with certain genetic changes while preserving healthy cells (Gonçalves et al., 2026). The most well-known example is the relationship between BRCA1/2 loss-of-function and poly ADP-ribose polymerase1 (PARP1) suppression in malignancies of the breast, ovarian, prostate, and pancreatic cancers. PARP compensates for the DNA-repair in cancer cells with deficient BRCA1/2 and first approved class of synthetic lethal drugs such as PARP inhibitors (e.g., Olaparib) disrupt PARP-mediated DNA repair thereby causing cytotoxicity in cancer cells (Prakash et al., 2015; Farmer et al., 2005). CRISPR-based functional genomics can rapidly identify new targets and thereby add to the existing and available synthetic lethal therapies.

### 3.7 Artificial intelligence in cancer genomics: solving complex data

**FIGURE 7:**
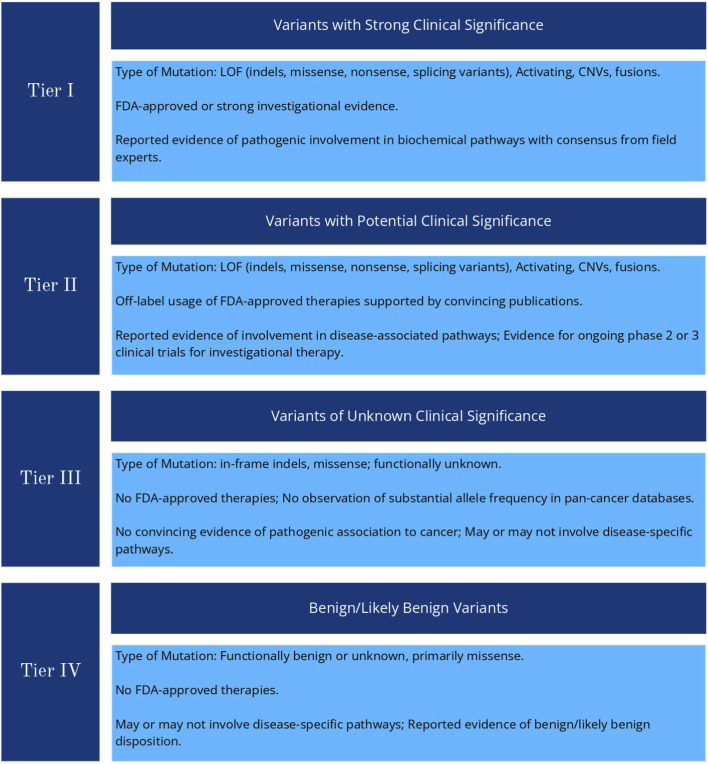
Genomic variant classification for actionability. Four-tier classification of somatic variants according to the AMP/ASCO/CAP guidelines. Based on the reported evidence of clinical significance, somatic variants are categorized either as variants with strong clinical significance, potential clinical significance, unknown clinical significance or benign. LOF, Loss of Function; CNV, Copy Number Variation.

Artificial intelligence (AI) and machine learning (ML) are becoming integral part of genomic research in precision and regenerative medicine (Ozcelik et al., 2024; Sharma et al., 2024). AI-driven approaches have been used to accelerate genomic analysis, enabling researchers to understand genetic variants without the risk of human error. AI, ML and deep learning (DL) have become increasingly important in the field of gene expression analysis (Shandhi and Dunn, 2022; Jones et al., 2017; Wang et al., 2024a). This is remarkably evident in technologies that use advanced neural network architectures to improve genomic analysis. AI, ML, DL-powered methods used in tools like burrows-wheeler aligner’s maximal exact matches (BWA-MEM) and spliced transcripts alignment to a reference (STAR) enhance the speed and accuracy of the genomic data analysis (Jung and Han, 2022; Athanasopoulou et al., 2025). High accuracy in genomic variant calling and annotation is crucial as errors often stem from low-complexity regions or mapping errors that lead to false positives, incorrect diagnoses, and misinterpretation of disease mechanisms. The classification of genomic variants into tiers (Figure 7) is based on the American college of medical genetics and genomics and association for molecular pathology guidelines. While simplified into tiers, the process of determining which tier a variant belongs to is complex, requiring expert, evidence-based judgment. DL-based tools such as Google’s DeepVariant and NVIDIA’s GPU-accelerated Parabricks leverage convolutional neural networks to improve accuracy, efficiency, and scalability in variant calling and data analysis (Abdelwahab and Torkamaneh, 2025; O’Connell et al., 2023). DeepTrio is a sophisticated DL-based variant caller that Google Health created as an expansion of DeepVariant with the purpose of examining genomic data from family trios which usually include a child and their two parents (Shadrina et al., 2024). DeepVariant is an open-source DL-based variant caller that analysis pileup image tensors of aligned reads using deep convolutional neural networks to provide highly accurate discovered variants (Poplin et al., 2018). DNAscope is a computationally efficient, highly accurate variant calling framework that can analyze both short- and long-read sequencing data (Hu et al., 2025). Hybrid and stand-alone estimation of small genomic variants (HELLO) is an open-source variant caller created to tackle the difficulties of identifying genomic variants of Indels and SNPs using PacBio, Illumina, and hybrid data (Ramachandran et al., 2021).

**FIGURE 8:**
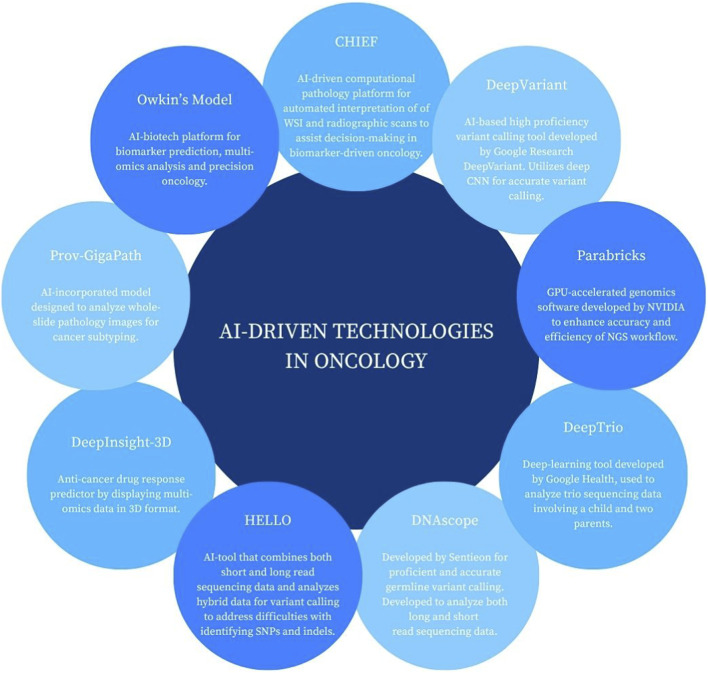
AI-driven technologies transforming oncology research and precision medicine. Summary of recent developments in artificial intelligence (AI)-based computational platforms and deep-learning tools currently advancing cancer genomics, pathology, and translational oncology.

Recent developments in AI, DL and ML have demonstrated impressive abilities for predicting treatment response in a variety of disorders including cancer (Figure 8). Computational techniques can characterize molecules numerically for ML algorithms to evaluate the generalizability of drug response models (An et al., 2022; Xia et al., 2022). Complex DL techniques have been used to forecast therapeutic responses in cancer cell lines; however, there are still issues such as overfitting small datasets (Chen and Zhang, 2022). Another recently reported DL-based method is DeepInsight-3D for predicting the response of anti-cancer drugs. DeepInsight-3D can capture intricate gene relationships by displaying multi-omics data in 3D format. This method provides predictions on tumor reactions to a particular anti-cancer drug when paired with patient-specific data (Sharma et al., 2023). The integration of AI, ML, and DL with NGS technologies has significantly advanced the interpretation of complex cancer genomic datasets. By enabling the rapid analysis of large-scale multi-omics data including genomic, transcriptomic, epigenomic, and proteomic, AI-driven approaches facilitate the identification of novel biomarkers, driver mutations, and molecular signatures associated with tumor initiation, progression, and therapeutic response (Wu Y. et al., 2025; Kant and Deepika, 2025). Furthermore, AI-based predictive models have demonstrated considerable potential in improving cancer diagnosis, risk stratification, and treatment selection thereby supporting the implementation of precision oncology (Srivastav et al., 2025; Li J. et al., 2026; Wang et al., 2025). Additionally, recent advances in AI foundation models for computational pathology have demonstrated the transformative potential of large-scale multimodal AI frameworks in cancer diagnosis, molecular profiling, and prognostic prediction. Owkin’s models, CHIEF (Clinical Histopathology Imaging Evaluation Foundation), Prov-GigaPath and DeepMind AI by google have been recently developed as cancer detection imaging AI models. Owkin is a NextGen AI company building AI-tools for biomedical breakthroughs. The Owkin model leverages multimodal deep learning frameworks to analyze decentralized patient datasets across institutions without directly sharing sensitive patient information. This approach has facilitated the development of predictive and prognostic models capable of identifying treatment-response signatures, molecular biomarkers, and clinically actionable targets in cancer. Owkin’s AI platforms have demonstrated utility in predicting histological response to neoadjuvant chemotherapy in triple-negative breast cancer, detecting microsatellite instability directly from histopathology images, and inferring transcriptomic features from whole-slide pathology images (Ogier du Terrail et al., 2023). Another recent AI based advancement with a focus on improving cancer diagnosis and precision oncology is CHIEF. This AI-driven computational pathology platform represents an emerging technology focused on improving cancer diagnosis and precision oncology through DL-based analysis of medical imaging and histopathological data. Convolutional neural network architectures and cloud-based machine learning infrastructure are used in CHIEF to analyze radiological and histopathological images for the detection and characterization of malignancies, including breast, lung, and colorectal cancers. CHIEF is trained on extensive histopathology datasets using self-supervised and weakly supervised learning approaches enabling extraction of biologically meaningful interpretations from digitized tissue sections. The platform integrates computational pathology with digital imaging workflows for automated interpretation of whole-slide images and radiographic scans to support clinical decision-making in biomarker-driven oncology. Notably, CHIEF has shown strong performance in pan-cancer detection, prediction of molecular alterations such as microsatellite instability and mutation status, survival prediction, and identification of tumor-specific genomic signatures directly from histopathological images (Wang X. et al., 2024; Zhang et al., 2026). Similarly, Prov-GigaPath is another AI- driven platform that represents a major advancement in computational pathology and multimodal cancer diagnostics. Developed through a collaboration between Microsoft, Providence Health System, and the University of Washington, Prov-GigaPath is a whole-slide pathology foundation model pretrained on approximately 1.3 billion pathology image tiles derived from more than 171,000 whole-slide images collected from over 30,000 patients across 31 tissue types. Prov-GigaPath has demonstrated excellent performance across multiple digital pathology applications, including cancer subtyping, biomarker and mutation predictions. Importantly, Prov-GigaPath significantly improved the prediction of clinically relevant genomic alterations, including mutations in EGFR, KRAS, TP53, and LRP1B, directly from histopathological images, highlighting its potential utility in precision oncology and AI-assisted companion diagnostics (Xu et al., 2024). As computational algorithms continue to evolve with increasing incorporation of DL, network-based modeling, and integrative multi-modal data analysis, AI is expected to further enhance our ability to decode tumor heterogeneity and uncover actionable therapeutic targets. Nevertheless, challenges such as data standardization, algorithm interpretability, model reproducibility, and ethical considerations related to data privacy must be addressed to ensure reliable clinical translation. Collectively, the convergence of AI with cancer genomics represents a transformative paradigm that will continue to reshape cancer research and accelerate the development of personalized diagnostic and therapeutic strategies.

## 4 Genomic innovations in cancer prevention

Although early detection and timely treatment represent a fundamental objective of modern medicine, achieving this goal in practice remains a highly complex and multifactorial challenge. Advances in genomics have transformed understanding of cancer initiation and progression enabling the development of strategies that move beyond early detection and treatment toward true prevention. By identifying genetic predispositions, early molecular alterations, and modifiable genomic risk factors, genomic approaches allow for personalized cancer prevention and risk reduction interventions tailored to individual genetic profiles (Beane et al., 2017). Polygenic risk scores (PRS) and germline genetic testing are two complementary genomic techniques that enhance cancer risk prediction enabling early intervention and precision preventative strategies.

### 4.1 Germline testing and polygenic risk scores

Tumor sequencing alone is insufficient to provide the comprehensive genetic knowledge required for cancer risk prediction (Liu and Stadler, 2021). The primary purpose of germline testing is to identify heritable mutations present in the DNA of all body cells passed from parents to offspring. These mutations often confer high risk in predisposed individuals to specific cancer types (Robson et al., 2015; Stadler et al., 2014). BRCA1/2 mutations in hereditary breast and ovarian syndrome are one example of the growing number of cancer susceptibility genes that both increase cancer risk and may guide cancer treatment (Ponti et al., 2023). The germline information can inform a wide range of clinical decisions, from prognosis to precision therapy selection, surgical decisions, reproductive choices, and clinical trial qualification. A hereditary cancer syndrome is a genetic propensity to specific cancers with early onset brought on by inherited pathogenic mutations in one or more genes. A particular hereditary cancer syndrome may be present in cases that obstetrician-gynecologists and other care providers frequently encounter such as ovarian, breast, colon, and endometrial cancer (Hereditary Cancer Syndromes and Risk Assessment: ACOG COMMITTEE OPINION, 2019). Common types of cancer syndromes are Lynch syndrome, Li-Fraumeni syndrome, Cowden syndrome and Peutz-Jeghers syndrome. Hereditary cancer risk assessment plays a critical role in identifying individuals and families who may have an elevated genetic predisposition to specific cancer types (Ashton-Prolla et al., 1992).

PRS have emerged as a powerful approach for quantifying inherited susceptibility to cancer by integrating the cumulative effects of common genetic variants across the genome (Wang et al., 2021). Investigating high-penetrance germline mutations (e.g., BRCA1/2) can risk-stratify a small subset of individuals whereas, PRS capture the combined influence of many low-penetrance single nucleotide polymorphisms (SNPs) identified through large-scale genome-wide association studies (GWAS) (Cross et al., 2022). PRS can stratify individuals based on their genetic predisposition to specific cancers by aggregating the weighted contribution of these variants into a single quantitative score (Wang et al., 2021). This quantitative PRS can be used to risk-stratify individuals with predisposition to breast, prostate, colorectal, and lung cancers. Recent studies have demonstrated that individuals in the highest PRS percentiles can exhibit several-fold increased disease risk compared with the general population highlighting the potential of PRS for population-level risk stratification and targeted screening strategies (Liu et al., 2025). In the context of precision oncology, PRS may complement traditional clinical and environmental risk factors to refine early detection efforts, guide preventive interventions, and inform personalized surveillance programs. However, challenges remain in the clinical implementation of PRS, including limited transferability across diverse populations, the need for large and ethnically representative genomic datasets, and the integration of polygenic risk models with other molecular and lifestyle factors. One of the major challenges is the substantial underrepresentation of diverse ancestral populations in GWAS and large-scale genomic databases. Most currently available PRS models have been developed predominantly using datasets derived from individuals of European ancestry leading to reduced predictive accuracy and limited transferability when applied to non-European populations (Sakaue et al., 2021). Differences in allele frequencies, linkage disequilibrium patterns, genetic architecture, and environmental interactions across populations can significantly affect PRS performance and may result in inaccurate risk estimation in underrepresented groups. This lack of diversity in genomic datasets raises important concerns regarding equity and inclusivity in precision oncology. The application of ancestry-biased PRS models may exacerbate existing healthcare disparities by limiting access to accurate genomic risk assessment among populations from Africa, Asia, Latin America, and other underrepresented regions (Martin et al., 2019). In addition, socioeconomic inequalities, limited access to genomic testing infrastructure, and variability in clinical implementation further complicate the global applicability of PRS-guided prevention strategies. Notably, efforts are underway for the development of ethnically diverse genomic datasets, trans-ancestry GWAS, and integrative multi-omic risk models to improve the accuracy, generalizability, and clinical utility of PRS across global populations (Nguyen et al., 2026). Overall, PRS is expected to play an important role in cancer risk prediction and preventive oncology as genomic datasets continue to expand with new analytical frameworks.

### 4.2 Population genomics and screening programs

Population genomic screening of adults has emerged as a strategy for the prevention of common diseases such as cancer among people with genetic conditions. Population genomics utilizes large-scale genomic data to understand the distribution of genetic variation across populations and its impact on disease risk, treatment response, and the development of prevention strategies. Integrating genomic data into population-level screening programs enables early cancer identification in individuals at risk thereby paving the way for precision public health (Fatumo et al., 2022). In cancer, population genomics plays a pivotal role in identifying hereditary cancer predisposition variants, population-specific allele frequencies, and ancestry-informed risk factors (Wang et al., 2024b). Researchers can uncover common pathogenic variants associated with familial cancers as well as low-penetrance variants contributing to polygenic risk by examining genomic data across diverse cohorts. These insights form the basis for developing genetic screening programs, risk prediction models, and precision prevention strategies tailored to different populations (Oak et al., 2020).

Lynch syndrome is a common inherited cancer condition caused by germline mutations in the mismatch repair (MMR) genes. It is most frequently linked to colorectal, uterine, stomach, ovarian, urinary tract, biliary tract, prostate, pancreas, brain, and skin cancers (Idos and Valle, 1993). The Amsterdam criteria II and revised Bethesda guidelines are critical clinical tools used to identify high-risk families and individuals for Lynch syndrome (Trujillo-Rojas et al., 2023). Lynch syndrome is an autosomal dominant condition that increases susceptibility to cancer and Amsterdam II criteria is proven to be efficient in identifying families at high risk (Peltomäki et al., 2023). These requirements state that a family must have three or more people with cancers linked to Lynch syndrome and at least one of whom must be a first-degree relative of the other two. These studies emphasize the significance of genetic screening, early detection, and careful monitoring in high-risk groups. Though genetic mechanisms influence malignancies, lifestyle factors such as obesity and hormone imbalances have a substantial impact on the development and progression of many cancers. Innovations in NGS and high-throughput omics technologies will aid in rapid population level genomic data generation from diverse groups which may further help in the efficient identification of new biomarkers for risk-stratification.

Human papillomavirus (HPV) and Epstein–Barr virus (EBV) are among the most well-established oncogenic viruses contributing to a significant global burden of virus-associated malignancies. Advances in genomic technologies including NGS, digital PCR, and cfDNA analysis have enabled detailed characterization of viral genomes, integration events, and host-virus interactions that drive carcinogenesis (Fernandes et al., 2020; Amponsah et al., 2026). Finding high-risk genotypes such as HPV16 and HPV18 and monitoring viral integration sites within the host genome are the main goals of HPV genomic surveillance. Oncogenes E6 and E7 are frequently overexpressed because of integration disruption of host tumour suppressor genes and viral regulatory areas (Islam et al., 2026; Yeo-Teh et al., 2018). Whole-genome and transcriptome sequencing have identified patterns of HPV integration associated with disease development in cervical, oropharyngeal, and anal malignancies (Minhas et al., 2023). Liquid biopsy based NGS or PCR methods that identify circulating HPV DNA offer a minimally invasive way to track disease recurrence, treatment response, and residual illness with real-time precision management (Parisi et al., 2025). Nasopharyngeal carcinoma, gastric cancer, and lymphoproliferative diseases are etiologically linked to EBV infection. Quantifying viral burden, identifying episomal versus integrated viral DNA, and analyzing viral gene expression patterns are the main goals of genomic monitoring of EBV (Yu et al., 2021). Strain variety, transcriptional programs, and co-evolution with host genomes that affect oncogenic potential and immune evasion are revealed by EBV whole-genome sequencing and metagenomic investigations (Xu et al., 2019). Integration of EBV genomics with host mutational and epigenetic data has revealed virus-host genomic interactions driving tumor heterogeneity and progression (Chung et al., 2025).

### 4.3 Precision prevention

Precision prevention represents a paradigm shift from generalized population-based disease prevention strategies toward individualized, risk-adapted interventions informed by genetic, molecular, environmental, and behavioral determinants. This strategy aims to identify individuals likely to develop diseases and take preventative measures, such as customized screening, lifestyle changes, chemoprevention, or surveillance techniques before clinical manifestation (Narimatsu et al., 2025). The use of pharmaceutical or nutritional treatments to prevent or delay the start of cancer based on genomic susceptibility refers to chemoprevention. Advances in germline and somatic genomics have enabled the development of targeted chemopreventive strategies tailored to an individual’s genetic and molecular risk profile. These chemopreventive strategies help in maximizing preventive efficacy while minimizing unnecessary exposure and potential adverse effects (Ren et al., 2025). Chemoprevention of cancer affects the development, progression, and metastasis of the disease. The goal of primary chemoprevention is to stop diseases from developing in the general population or in populations that are particularly at risk. Patients with a specific kind of tumour or precancerous lesion that could progress to invasive cancer are the target of secondary chemoprevention. The goal of tertiary chemoprevention is to prevent secondary tumors or cancer recurrence (Penny and Wallace, 2015). Individuals carrying high-penetrance germline mutations benefit from targeted chemoprevention strategies. Selective estrogen receptor modulators (SERMs) such as tamoxifen or raloxifene are available to carriers of the BRCA1/2 mutation and dramatically lower the risk of estrogen receptor-positive breast cancer (Alwashmi et al., 2025). Research has shown that daily aspirin consumption of patients with Lynch syndrome have a lower risk of colorectal cancer (Burn et al., 2011).

## 5 Genomic innovations in cancer treatment

Personalized medicine has become a groundbreaking strategy for the treatment of cancer in contemporary healthcare. In contrast to conventional approaches that rely on generalized treatment plans, personalized medicine uses genomic data to customize treatments to a patient’s genetic profile (Molla and Bitew, 2024). Advances in NGS technologies enabled accurate diagnosis and focused treatment approaches that enhance patient outcomes while reducing side effects (Ghoreyshi et al., 2025). Additionally, identifying genetic variants by NGS has laid the foundation for numerous targeted therapies based clinical trials (Table 2).

**TABLE 2:** Clinical trials of genomics-guided cancer targeted therapeutics supported by PubMed-indexed literature.

Trial number	Status	Cancer type	Genomic alteration targeted	Key findings
NCT04414150	Completed	Solid tumors	Anti LAG-3 antibody SHR-1802	A tolerable safety profile and preliminary antitumor activity in patients was demonstrated by SHR-1802 in advanced solid tumors (Deng et al., 2023)
NCT04784715	Ongoing	Breast cancer	HER2	Trastuzumab deruxtecan plus pertuzumab led to a significantly lower risk of progression or death when used as first-line treatment for HER2-positive advanced or metastatic breast cancer (Tolaney et al., 2025)
NCT02975934	Completed	Prostate cancer	PARP	The duration of imaging-based progression-free survival was significantly longer with rucaparib than with a control medication among patients who had metastatic, castration-resistant prostate cancer with a BRCA alteration (Fizazi et al., 2023)
NCT04487080	Ongoing	NSCLC	EGFR	Amivantamab-lazertinib led to significantly longer overall survival among participants with previously untreated EGFR-mutated advanced NSCLC than osimertinib but was associated with an increased risk of adverse events of grade 3 or higher (Yang et al., 2025)
NCT06533579	Ongoing	CD19-positive hematologic malignancies	GP101	This is an ongoing Phase 1/2, first-in-human, open-label, dose-escalating trial designed to assess the safety and efficacy of VNX-101 in patients with relapsed or refractory CD19-positive hematologic malignancies (Currier et al., 2025)
NCT03310879	Ongoing	Metastatic solid tumors	CCND1, CCND2, CCND3, CDK4, or CDK6	An ongoing phase ii study of the CDK4/6 inhibitor abemaciclib in patients with solid tumors harboring genetic alterations in genes encoding d-type cyclins or amplification of CDK4 or CDK6 (Kalinsky et al., 2025)
NCT06136897	Ongoing	Malignant solid neoplasm	HER2	This ongoing phase II MATCH treatment trial tests how well trastuzumab and pertuzumab work in treating patients with HER2-amplified non-breast, non-gastric/gastroesophageal junction, and non-colorectal cancers (Connolly et al., 2024)
NCT06946615	Ongoing	Colorectal Cancer	CLDN18	This ongoing study aims to evaluate the safety and feasibility of combining Claudin18.2-targeted CAR-T cells with CAR-engineered dendritic cells (CAR-DCs) in patients with advanced colorectal cancer. The primary goal is to determine the maximum tolerated dose, anti-tumor efficacy, and survival outcomes of CAR-DCs when given with CAR-T cells during the dose-escalation phase (Khelwatty et al., 2025)
NCT03020017	Completed	Recurrent Glioblastoma or Gliosarcoma	Bcl2L12	The study describes spherical nucleic acids (SNAs) as a novel nanotherapeutic platform consisting of a nanoparticle core densely coated with radially arranged synthetic oligonucleotides. Early-phase clinical trials in glioblastoma and solid tumors demonstrated that SNAs are safe, capable of penetrating the brain, and effective in modulating tumor gene expression and immune activation. This highlights clinical findings, next-generation SNA design strategies, and future directions for optimizing their anti-cancer potential (Kumthekar et al., 2021)
NCT06140732	Ongoing	Advanced Chordoma	BRAF	This ongoing phase II trial evaluates Camrelizumab combined with Apatinib treatment in patients with chordoma with metastasis (Liu et al., 2020)
NCT04283006	Ongoing	Relapsed or Refractory Lymphoid Malignancies	CD20 and CD22	The purpose of the study is to evaluate the safety and effectiveness of CD20/ CD22 dual-target CAR T-cell therapy and to provide clinical basis and experience for CAR T-cell technology in the treatment of clinical malignant haematological diseases (Shah and Sokol, 2021)
NCT06972576	Ongoing	Non-Small Cell Lung Cancer	EPHA2	Single-arm clinical study designed to evaluate the safety and preliminary efficacy of EphA2-targeted CAR-DC combined with CAR-T cell therapy in patients with non-small cell lung cancer (Rafii et al., 2025)
NCT02322853	Terminated	Breast Cancer	p38 MAPK	LY2228820, a selective inhibitor of p38 MAPK (α and β isoforms), was investigated for its potential to enhance endocrine therapy by blocking MAPK signaling and reducing phosphorylation of MAPKAP-K2. Trial was terminated due to lack of participant enrollment (Patnaik et al., 2016)
NCT01176669	Completed	Triple-Negative Breast Cancer	VEGF	Study demonstrated substantial antitumor activity and manageable toxicities of apatinib in patients with advanced breast cancer (Fan et al., 2014)
NCT01653561	Completed	non-triple-negative metastatic breast cancer	VEGF	Apatinib is a tyrosine kinase inhibitor targeting vascular endothelial growth factor receptor (VEGFR), and its anti-angiogenesis effect has been viewed in preclinical tests. Apatinib exhibited objective efficacy in heavily pretreated, metastatic non-triple-negative breast cancer with manageable toxicity underlining treatment opportunity in breast cancer patients with high angiogenesis dependency (Hu et al., 2014)
NCT04430530	Unknown status	CD19-negative B Malignancies	T cells	This study evaluated safety and efficacy of 4th generation chimeric antigen receptor gene-modified T cells (4SCAR-T) targeting CD19-negative B-ALL that express alternative surface antigens such as CD22, CD10, CD20, CD38, and CD123, as many patients relapse after anti-CD19 immunotherapy (Clubb et al., 2023)
NCT06006390	Ongoing	CEA-positive advanced malignant tumors	CEA-CAR-T	This study evaluates the safety and efficacy of CEA-targeted CAR-T cells in patients with CEA-positive advanced malignant tumors In NCSLC, responders exhibited a higher percentage of NK cells in a less exhausted state, characterized by reduced activity of immunosuppressive pathways (e.g., TGF-β signaling and hypoxia) and lower expression of stress-associated genes (e.g., DUSP1, HSPB1) (Holtermann et al., 2024)
NCT06158139	Ongoing	Pancreatic ductal adenocarcinoma	CD276	This gene therapy research study aims to evaluate the safety and tolerability of a new treatment called autologous T lymphocyte chimeric antigen receptor cells targeting the B7-H3 antigen (iC9.CAR.B7-H3 T cells) in patients with pancreatic ductal adenocarcinoma that has relapsed after standard therapy (Guirguis et al., 2025)
NCT04660929	Ongoing	HER2 positive cancer	HER2	Phase 1, first in human study of adenovirally transduced autologous macrophages engineered to contain an anti-HER2 chimeric antigen receptor in subjects with HER2 overexpressing solid tumors (Reiss et al., 2025)
NCT04196413	Ongoing	Diffuse midline gliomas	GD2	This early-phase clinical trial evaluates the feasibility and safety of GD2-targeted CAR-T cell therapy in children and adults with H3K27M-mutant diffuse midline glioma. The study examines whether patients’ own T cells can be successfully manufactured, safely administered at increasing doses, and delivered through different routes, including repeat intracerebroventricular infusions (Monje et al., 2025; Majzner et al., 2022)
NCT02208362	Ongoing	Glioblastoma	IL13Rα2	This phase I trial tests the safety and optimal dose of IL13Rα2-targeted CAR T-cell therapy in patients with recurrent or treatment-resistant malignant glioma. Patients’ own T cells are genetically engineered to recognize glioma cells and are delivered directly into the tumor or brain ventricles using different routes. The study focuses on identifying a safe dose and delivery method while exploring early signs of treatment response and immune activity in the brain (Brown et al., 2024; Brown et al., 2016)
NCT04581473	Ongoing	Gastric and Pancreatic cancers	CLDN18.2	This open, multicenter Phase Ib/II trial tests the safety, optimal dose, and effectiveness of CT041, a Claudin18.2-targeted autologous CAR T-cell therapy, in patients with advanced gastric/gastroesophageal junction adenocarcinoma and pancreatic cancer who have failed prior treatments. The study uses dose escalation followed by expansion and then confirms efficacy and safety in Phase II (Qi et al., 2024; Qi et al., 2025a; Qi et al., 2023)
NCT01869166	Completed	Advanced EGFR-positive solid tumors	EGFR	Study tests the safety and feasibility of EGFR-targeted CAR T-cell therapy in patients with advanced, EGFR-positive solid tumors. Patients’ own T cells are genetically modified to recognize EGFR on cancer cells and infused back to trigger an immune response with monitoring side effects, persistence of the CAR T cells, and early signs of antitumor activity (Guo et al., 2018)
NCT02706392	Terminated due to slow accruals	CLL, MCL, ALL, stage IV NSCLC, and TNBC	ROR1	The Phase I trial evaluated the safety and optimal dosing of ROR1-targeted CAR-T cells in patients with ROR1-positive cancers, including CLL, MCL, ALL, stage IV NSCLC, and TNBC. The study showed that TNBC patients tolerated escalating CAR-T cell doses without severe cytokine release syndrome or neurotoxicity. Some patients achieved stable disease, and one showed a partial response after a second infusion. However, despite evidence of CAR-T cell expansion in peripheral blood, tumor infiltration and persistence were limited, and no consistent tumor regression was observed. These findings suggest that an immunosuppressive TME may impair CAR-T cell function by reducing cytokine production and promoting inhibitory receptor expression (Srivastava et al., 2021; Osorio-Rodríguez et al., 2023)
NCT05947487	Ongoing	CD70-positive advanced or metastatic solid tumors	CD70	This single-center, open-label Phase I/II study evaluates the safety, optimal dose, and preliminary efficacy of autologous CD70-targeted CAR-T cell therapy in patients with CD70-positive advanced or metastatic solid tumors. The Phase I portion uses a 3 + 3 dose-escalation design to assess safety across three increasing dose levels, while the Phase II expansion enrolls additional patients at the recommended dose to further evaluate antitumor activity. Overall, the trial aims to determine whether CD70-CAR-T cells can be safely administered and show therapeutic benefit in solid tumors, building on promising preclinical results (Kasap et al., 2024)
NCT05812326	Completed	Breast Cancer	MUC1	This phase I clinical trial evaluated the safety and tolerability of PD-1–knockout, MUC1-targeted CAR-T cells in patients with advanced breast cancer. Patient-derived T cells were engineered to express a MUC1-specific CAR and then edited using CRISPR-Cas9 to remove PD-1, aiming to enhance antitumor activity. Twelve patients received one or more cycles of escalating CAR-T cell doses. The treatment was well tolerated, with mostly mild to moderate adverse events, no severe cytokine release syndrome, no neurotoxicity, and no dose-limiting toxicities. Five patients achieved stable disease, suggesting preliminary disease control. Overall, the study demonstrates that PD-1–disrupted MUC1 CAR-T cell therapy is safe and feasible, with early signs of potential clinical benefit (Gorodetska et al., 2025)
NCT04044768	Ongoing	Synovial sarcoma or myxoid round cell liposarcoma	MAGEA4	This phase 2, open-label trial (SPEARHEAD-1) evaluated the efficacy and safety of afamitresgene autoleucel (afami-cel), a T-cell receptor–based therapy, in patients with advanced synovial sarcoma or myxoid round cell liposarcoma whose tumors expressed MAGE-A4 and carried the HLA-A*02 allele. Heavily pretreated patients received a single infusion of afami-cel following lymphodepleting chemotherapy. The study demonstrated a meaningful overall response rate, with durable tumor responses particularly in synovial sarcoma, while showing a manageable safety profile characterized mainly by cytokine release syndrome and cytopenia, and no treatment-related deaths. Overall, the results support the effectiveness of TCR-based immunotherapy in selected solid tumors and justify further expansion of this therapeutic approach (D’Angelo et al., 2024)
NCT04729543	Ongoing	Melanoma and head-and-neck squamous cell carcinoma	MC2	This first-in-human Phase I/II trial evaluates the safety and preliminary efficacy of MAGE-C2/HLA-A2–specific TCR-engineered T cells in patients with advanced melanoma and head-and-neck squamous cell carcinoma. The study uses genetically modified T cells with a “young” phenotype to target the cancer germline antigen MAGE-C2, which is highly expressed in these tumors but not in normal tissues. Patients receive epigenetic pretreatment to enhance tumor antigen expression, followed by TCR T-cell infusion without prior chemotherapy, with the goal of assessing safety and antitumor activity (Kunert et al., 2016)
NCT06224738	Ongoing	HER2+ gastric cancer	HER2	This ongoing clinical trial tests the safety and early efficacy of HER2-targeted CAR macrophage (CAR-M) therapy in patients with advanced HER2-positive gastric cancer. Autologous macrophages are genetically engineered to recognize HER2 and infused intraperitoneally to induce antitumor effects, with the goal of assessing the feasibility of CAR-macrophage therapy in solid tumors (Dong et al., 2023)
NCT05430945	Completed	Multiple myeloma	BCMA	This clinical trial evaluates the safety and effectiveness of BCMA-targeted CAR-T cell therapy in patients with relapsed or refractory multiple myeloma, while expanding patient numbers to better capture rare and delayed toxicities. In parallel, the study investigates ctDNA as a non-invasive biomarker to predict treatment response and prognosis. The findings show that higher tumor burden, reduced CAR-T cell expansion, and elevated ctDNA levels. Additionally, specific high-risk genetic mutations are associated with poorer outcomes, leading to the development of a ctDNA-based risk model to help predict response to anti-BCMA CAR-T therapy (Chen et al., 2024)
NCT03233204	Completed	Solid tumors and non-Hodgkin lymphomas	DDR genes	Pediatric Molecular Analysis for Therapy Choice (MATCH) study evaluated the safety and efficacy of the PARP inhibitor olaparib in children and adolescents with treatment-refractory solid tumors harboring DNA damage repair gene alterations. Although patients were molecularly matched based on tumor sequencing, most identified alterations were germline and monoallelic, and few tumors showed features predictive of sensitivity to PARP inhibition. Olaparib was well tolerated with minimal toxicity, but no objective responses were observed, and the study was closed early due to low accrual and limited clinical benefit (Glade et al., 2024)
NCT04266301	Terminated	Chronic Myelomonocytic Leukemia-2	Tim-3	This Phase III trial evaluated sabatolimab plus azacitidine versus azacitidine alone in higher-risk MDS and CMML-2 patients ineligible for intensive therapy. Although earlier studies showed good tolerability, the Phase II and III trials failed to meet primary efficacy endpoints, leading to program termination due to limited clinical benefit rather than safety concerns (Zeidan et al., 2023)
NCT04082364	Completed	Gastric cancer or gastroesophageal Junction cancer	HER2	This Phase 2/3 randomized, open-label MAHOGANY trial evaluates margetuximab in combination with immune checkpoint inhibitors and chemotherapy in patients with HER2-positive gastric or gastroesophageal junction cancer. Part A assesses the safety and efficacy of margetuximab plus retifanlimab, while part B compares multiple combination regimens against standard therapy. Based on encouraging results, the trial aims to determine whether these combinations can improve outcomes in treatment-naïve HER2-positive patients (Catenacci et al., 2021)
NCT02744287	Suspended	Metastatic pancreatic or castration-resistant prostate cancer	PSCA	This Phase I, multi-institutional dose-escalation trial evaluated the safety and preliminary efficacy of BPX-601, a PSCA-targeted GoCAR-T® cell therapy activated by rimiducid, in patients with metastatic pancreatic and castration-resistant prostate cancer. While CAR-T cell expansion, persistence, and tumor infiltration were observed and some prostate cancer patients showed clinical responses, dose-limiting toxicities and treatment-related deaths at the highest dose led to early study termination (Stein et al., 2024)
NCT04186520	Ongoing	B cell malignancies	CD19 and CD20	This study investigated the functional quality of bispecific CD19/CD20 CAR T cells designed to reduce relapses caused by antigen loss after single-target CAR-T therapy. Single-cell proteomic analysis revealed that these CAR T cells showed high polyfunctionality and strong cytokine activity when stimulated with either CD19 or CD20. The results suggest that LV20.19 CAR T cells may maintain antitumor activity even when CD19 expression is reduced, supporting their potential to prevent antigen-escape–mediated relapse (Zurko et al., 2022; Shah et al., 2025)
NCT04684459	Ongoing	Solid tumors	HER2	This Phase I study evaluates the safety and preliminary efficacy of dual-targeting HER2/PD-L1 CAR-T cells in patients with HER2-positive solid tumors. By converting the inhibitory PD-L1 signal in the TME into an activating signal, the engineered CAR-T cells are designed to enhance tumor killing and persistence. Patients receive the modified T cells through intravenous or cavity infusion to assess feasibility, safety, and early antitumor activity (Ma et al., 2022)
NCT01352286	Completed	Multiple myeloma	NY-ESO-1	This study evaluated autologous T cells engineered to express an affinity-enhanced NY-ESO-1c259 T-cell receptor (SPEAR T cells) in 25 patients with relapsed or high-risk multiple myeloma who were HLA-A*02:01-positive and expressed NY-ESO-1 and/or LAGE-1. Treatment was well tolerated, with no cytokine release syndrome or fatal serious adverse events reported. The engineered T cells expanded *in vivo*, trafficked to bone marrow, persisted, and demonstrated tumor-specific activity, indicating promising antitumor efficacy in patients (Stadtmauer et al., 2019)
NCT00509288	Ongoing	Mesothelioma or mesothelin expressing cancers	Mesothelin	This study examined the fate of gene-engineered T cells used in adoptive cell-transfer therapy by comparing gene expression profiles of infused TCR-engineered lymphocytes with those persisting in circulation one-month post-infusion. Differentially expressed immune-related genes, with post-infusion cells showing reduced expression of stimulatory genes and increased expression of inhibitory genes was identified (Abate-Daga et al., 2013)
NCT05502237	Ongoing	NSCLC	TIGIT	This STAR-121 Phase III trial evaluates the efficacy and safety of dual immune checkpoint inhibition with domvanalimab (anti-TIGIT) and zimberelimab (anti–PD-1) plus chemotherapy versus standard pembrolizumab plus chemotherapy as first-line treatment for metastatic NSCLC without actionable gene alterations. Approximately 720 untreated patients are randomized to compare progression-free survival and overall survival, with secondary endpoints assessing response, safety, quality of life, and exploratory biomarker analyses (Rodriguez-Abreu et al., 2024)
NCT04374305	Ongoing	Vestibular schwannomas, non-vestibular schwannomas	NF2	This Phase II basket trial evaluated the safety and efficacy of brigatinib in patients aged 12 years or older with NF2-related schwannomatosis and progressive tumors. Treatment with oral brigatinib led to radiographic responses and reduced tumor growth across multiple tumor types particularly meningiomas and nonvestibular schwannomas, along with improvements in hearing and pain. The therapy was well tolerated, with no severe treatment-related adverse events, demonstrating clinical benefits in this difficult-to-treat population (Plotkin et al., 2024)
NCT01362296	Completed	NSCLC	KRAS	This Phase II randomized trial compared the MEK inhibitor trametinib with docetaxel in patients with previously treated KRAS-mutant non-small cell lung cancer. The study found no significant difference in progression-free survival, overall survival, or response rates between the two treatments and was stopped early due to futility. Overall, trametinib demonstrated similar efficacy to standard chemotherapy but did not provide a clear clinical advantage in this patient population (Blumenschein et al., 2015)

### 5.1 Targeted therapy and companion diagnostics

Targeted and immunotherapy have revolutionized cancer treatment by leveraging individual patients unique genetic makeup and immune system. Cancer care has been shifted from a one-size-fits-all approach to more precise and personalized treatments over the past decade. Accumulating evidence about genetic variants such as point mutations, small insertion and deletions and gene fusions has encouraged many industries and start-ups to develop customized gene panels for NGS based validation of tumors (Table 3). These commercially available NGS panels will be modified and adjusted as per the growing genomic evidence in the field of cancer research. Companion diagnostic (CDx) tests have been developed to guide clinical decision making by identifying patients most likely to benefit from specific therapeutic strategies, including targeted therapies, immunotherapies, and enrollment in clinical trials (Andrews et al., 2025). A key feature of a CDx test is its ability to accurately and reliably identify patients that are most likely to benefit from a specific FDA-approved therapy. To ensure clinical validity and utility, CDx tests undergo extensive analytical and clinical validation followed by rigorous regulatory review by the US-FDA prior to market authorization. These tests are approved for use in conjunction with a specific therapeutic agent or class of therapeutic agents to guide treatment selection. Tests that meet the FDA’s stringent regulatory standards are formally authorized as CDx (Valla et al., 2021). Numerous CDx tests have been approved by FDA that can identify variants in curated panels of genes in the DNA/RNA isolated from either tumor tissue biopsy or liquid biopsy (Table 4). However, differences in sensitivity and specificity of tissue versus liquid biopsies, individual and combined diagnostic yield of DNA and RNA sequencing used in CDx tests are of great importance. Although tissue biopsy based molecular profiling remains the current gold standard for cancer diagnosis and genomic characterization, liquid biopsy has significantly expanded the ability to characterize tumor biology in real time (Vaidyanathan et al., 2018). Liquid biopsy demonstrates excellent specificity for many clinically actionable mutations; however, tissue biopsy generally retains superior sensitivity for low-frequency variants and remains necessary for histopathological confirmation and assessment of spatial tumor architecture (Vaidyanathan et al., 2018). Consequently, these technologies are increasingly viewed as complementary rather than mutually exclusive approaches in precision oncology. Integration of tissue-based and liquid biopsy profiling in CDx may substantially improve genomic characterization, resistance monitoring, and treatment stratification across multiple cancer types. Further, diagnostic yield of DNA versus RNA-seq contributes majorly in CDx assays. DNA/RNA-seq represent complementary genomic approaches that significantly contribute to the development and implementation of CDx in precision oncology (Keller-Evans et al., 2026). DNA-based NGS assays primarily identify genomic alterations such as SNPs, Indels, copy number alterations, and structural rearrangements that may serve as actionable biomarkers for targeted therapy selection. Consequently, DNA sequencing forms the foundation of many currently approved companion diagnostic platforms used for identifying therapeutically relevant alterations in genes such as EGFR, BRAF, KRAS, and BRCA1/2. However, DNA-seq alone may not fully capture the functional transcriptional consequences of genomic alterations or identify clinically relevant fusion transcripts with sufficient sensitivity (Keller-Evans et al., 2026). In this context, RNA-seq has emerged as a powerful complementary technology that improves the clinical yield of CDx by directly evaluating gene expression, alternative splicing, and transcriptionally active fusion events. RNA-seq demonstrates particular clinical utility in detecting actionable gene fusions involving ALK, ROS1, RET, NTRK, and FGFR, many of which may be missed or incompletely characterized by DNA-based approaches. As CDx continues to evolve, integrating genomics, transcriptomics, and epigenomics are expected to substantially improve the clinical yield of sequencing.

**TABLE 3:** Commercially available genomic based tests.

Manufacturer	Name of the test	Name of the technology	References
Tempus Labs, Inc	xT CDx	NGS	Beaubier et al. (2019)
OmniSeq	OmniSeq Comprehensive®	NGS	Conroy et al. (2021)
Thermo Fisher Scientific	Oncomine DX Target Test™	NGS	Qi et al. (2025b)
NantHealth	Omics Core (SM)	WES	Zhong et al. (2021)
NYU Langone Medical Center	NYU Langone Genome PACT assay	NGS	Vasudevaraja et al. (2024)
ACT Genomics	ACTOnco	NGS	Jan et al. (2022)
Illumina	TruSight™ Oncology	NGS	​
	Ion AmpliSeq™ Comprehensive Cancer Panel	NGS	Tsongalis et al. (2014)
	TruSeq® Amplicon Panel	NGS	Fang et al. (2013)
Paradigm	Paradigm Cancer Diagnostic (PcDx™) Panel	NGS	Weiss et al. (2015)
PathGroup	SmartGenomics™	NGS, cytogenomic array, other technologies	Stenzinger et al. (2024)
Caris Molecular Intelligence through Caris Life Sciences	Tumor profiling service	Multiple technologies	Spizzo et al. (2019)
Knight Diagnostic Labs	GeneTrails® Solid Tumor Panel	Multiplex PCR	Mitri et al. (2018)
Foundation Medicine	FoundationOne®CDx test (F1CDx)	NGS	Milbury et al. (2022)
Illumina	Praxis extended RAS Panel	NGS	Udar et al. (2020)
Myriad Genetics	myRisk hereditary panel	NGS	Hayashi et al. (2022)
Exact Sciences	OncoExTra	NGS: Whole-exome and Whole – transcriptome sequencing	De La et al. (2025)
NEO Genomics	NEO PanTracer LBx	NGS	Pipinikas et al. (2025)
Thermo Fisher Scientific	Oncomine DX express Test™	NGS	Espinoza et al. (2024)
Memorial Sloan Kettering Cancer Center	MSK-IMPACT™	NGS	Cheng et al. (2015)

**TABLE 4:** Genetics based CDx Panels approved by FDA and other agencies.

Indication	Name of the panel	Biomarkers	Targeted therapeutics	References
Foundation Medicine, Inc				
NSCLC	FoundationOne CDx and FoundationOne Liquid CDx	EGFR: Exon 19 deletions, Exon 21 L858R, Exon 20 insertions, T790M	Tarceva (erlotinib), Tagrisso (osimertinib), Iressa (gefitinib) Gilotrif (afatinib), Vizimpro (dacomitinib), Lazcluze (Lazertinib)	Milbury et al. (2022), Woodhouse et al. (2020)
		ALK rearrangements	Alunbrig (brigatinib), Alecensa (alectinib), Xalkori (crizotinib), Zykadia (ceritinib), Alecensa (alectinib)	Milbury et al. (2022), Woodhouse et al. (2020)
		BRAF V600E	Tafinlar (dabrafenib) in combination with Mekinist (trametinib) or Braftovi (encorafenib) in combination with Mektovi (binimetinib)	Milbury et al. (2022), Woodhouse et al. (2020)
		MET exon 14 skipping	Tabrecta (capmatinib)	Milbury et al. (2022), Woodhouse et al. (2020)
		ROS1 fusions	Rozlytrek (entrectinib)	Milbury et al. (2022), Woodhouse et al. (2020)
Breast cancer	FoundationOne CDx	HER2 amplification	Herceptin (trastuzumab), Perjeta (pertuzumab), Kadcyla (ado-trastuzumab emtansine)	Milbury et al. (2022), Woodhouse et al. (2020)
		PIK3CA, AKT1, and PTEN alterations	Truqap (capivasertib) in combination with Faslodex (fulvestrant)	Milbury et al. (2022), Woodhouse et al. (2020)
	FoundationOne CDx and FoundationOne Liquid CDx	PIK3CA variants including C420R, E542K, E545A, E545D, E545G, E545K, Q546E, Q546R, H1047L, H1047R, and H1047Y	Piqray (alpelisib) or ItovebI (inavolisib) in combination with palbociclib and fulvestrant	Milbury et al. (2022), Woodhouse et al. (2020)
Cholangiocarcinoma	FoundationOne CDx	FGFR2 fusions and select rearrangements	Pemazyre (pemigatinib)	Milbury et al. (2022), Woodhouse et al. (2020)
Colorectal Cancer	FoundationOne CDx	KRAS wild type (absence of mutations in codons 12, 13 and absence of mutations in exons 2, 3, and 4)	Erbitux (cetuximab) and Vectibix (panitumumab)	Milbury et al. (2022), Woodhouse et al. (2020)
	FoundationOne Liquid CDx	BRAF V600E	Braftovi (encorafenib) in combination with cetuximab	Milbury et al. (2022), Woodhouse et al. (2020)
Glioma	FoundationOne CDx	BRAF V600 mutations and BRAF fusions	Ojemda (tovorafenib)	Milbury et al. (2022), Woodhouse et al. (2020)
Melanoma	FoundationOne CDx	BRAF V600 mutations	Mekinist (trametinib) or Tecentriq (atezolizumab) in combination with Cotellic (cobimetinib) and Zelboraf (vemurafenib)	Milbury et al. (2022), Woodhouse et al. (2020)
Prostate Cancer	FoundationOne CDx	Homologous recombination repair (HRR) genes including ATM, BRCA1, BRCA2, BARD1, BRIP1, CDK12, CHEK1, CHEK2, FANCL, PALB2, RAD51B, RAD51C, RAD51D and RAD54L alterations	Lynparza (olaparib)	Milbury et al. (2022), Woodhouse et al. (2020)
	FoundationOne CDx and FoundationOne Liquid CDx	BRCA1, BRCA2 alterations	Lynparza (olaparib) in combination with abiraterone. Akeega (niraparib + abiraterone acetate). Rubraca (rucaparib)	Milbury et al. (2022), Woodhouse et al. (2020)
Ovarian Cancer	FoundationOne CDx	BRCA1, BRCA2 alterations	Lynparza (olaparib)	Milbury et al. (2022), Woodhouse et al. (2020)
Ovarian Cancer	FoundationFocus CDxBRCA Assay	BRCA1, BRCA2 alterations	Rubraca (rucaparib)	Ford et al. (2020)
Solid tumors	FoundationOne CDx	TMB ≥ 10 mutations per megabase	Keytruda (pembrolizumab)	Milbury et al. (2022)
	FoundationOne CDx	Microsatellite instability-High (MSI-H)	Keytruda (pembrolizumab)	Milbury et al. (2022)
	FoundationOne CDx	RET fusions	Retevmo (selpercatinib)	Milbury et al. (2022)
	FoundationOne CDx and FoundationOne Liquid CDx	NTRK1, NTRK2, and NTRK3 fusions	Rozlytrek (entrectinib) and Vitrakvi (larotrectinib)	Milbury et al. (2022), Woodhouse et al. (2020)
Abbott Molecular, Inc.				
Acute Myeloid Leukemia	Abbott RealTime IDH1	IDH1 gene variants including R132C, R132H, R132G, R132S and R132L	Tibsovo (Ivosidenib). Rezlidhia (Olutasidenib)	Ragon and DiNardo (2017)
​	Abbott RealTime IDH2	R140Q, R140L, R140G, R140W, R172K, R172M, R172G, R172S and R172W mutations of IDH2 gene	Idhifa (Enasidenib)	Ragon and DiNardo (2017)
B-cell chronic lymphocytic leukemia	Vysis CLL FISH Probe Kit	17p deletion of TP53 gene	Venclexta™ (Venetoclax)	Venetoclax Approved for CLL (2016)
Resolution Bioscience, Inc.				
NSCLC	Agilent Resolution ctDx FIRST assay	KRAS G12C	Krazati (adagrasib)	Paweletz et al. (2023)
Myriad Genetic Laboratories, Inc.				
Ovarian, breast, pancreatic and prostate cancers	BRACAnalysis CDx	BRCA1 and BRCA2 mutations	Lynparza (olaparib), Talzenna (talazoparib), Rubraca (rucaparib)	Hong et al. (2021)
Ovarian Cancer	Myriad myChoice CDx	Deleterious or suspected deleterious mutations in BRCA1 and BRCA2 genes and/or positive Genomic Instability Score	Lynparza (olaparib)	Fountzilas et al. (2023)
Roche Molecular Systems, Inc.				
NSCLC	Cobas EGFR Mutation Tests v1 and v2	Exon 19 deletion or exon 21 L858R substitution mutation and T790M	Tarceva (erlotinib), Tagrisso (osimertinib), Iressa (gefitinib) Gilotrif (afatinib), Vizimpro (dacomitinib), Lazcluze (Lazertinib), Gilotrif (afatinib)	Claus et al. (2024)
Melanoma	Cobas 4800 BRAF V600 Mutation Test	BRAF V600K and V600E	Zelboraf (vemurafenib). Cotellic (cobimetinib) in combination with Zelboraf (vemurafenib)	Mourah et al. (2015)
Colorectal Cancer	Cobas KRAS Mutation Test	Mutations in codons 12 and 13 of KRAS gene	Erbitux (cetuximab). Vectibix (panitumumab)	Sharma et al. (2016)
Follicular Lymphoma	Cobas EZH2 Mutation Test	EZH2 gene variants including Y646N, Y646F or Y646X (Y646H, Y646S, or Y646C), A682G, and A692V	Tazverik (tazemetostat)	Shyu et al. (2023)
EntroGen, Inc.				
Colorectal Cancer	CRCDx RAS Mutation Detection Assay Kit	KRAS wild-type biomarkers (the absence of mutations in exons 2, 3, or 4) and NRAS wild-type biomarkers (the absence of mutations in exons 2, 3, or 4)	Vectibix (panitumumab)	Kang et al. (2024)
Guardant Health, Inc.				
NSCLC	Guardant360 CDx	EGFR exon 19 deletions, EGFR exon 21 L858R, and T790M. EGFR exon 20 insertions	Tagrisso (osimertinib). Rybrevant (amivantamb)	Olsen et al. (2022)
		KRAS G12C	Lumakras (sotorasib)	Bauml et al. (2022)
		ERBB2 Activating Mutations (SNVs And Exon 20 Insertions)	Enhertu	Qi et al. (2025c)
Breast cancer	Guardant360 CDx	ESR1 missense mutations between codons 310 and 547. ESR1 E380, V422del, S463, L469, L536, Y537, and D538 mutations	Orserdu (elacestrant). Imlunestrant (Inluriyo)	Abbasi et al. (2023)
Biocartis US, Inc.				
Colorectal Cancer	Idylla CDx MSI Test	Microsatellite instability-High (MSI-H) biomarkers including ACVR2A, BTBD7, DIDO1, MRE11, RYR3, SEC31A and SULF2	Herceptin (trastuzumab)	Andre et al. (2026)
Invivoscribe Technologies, Inc.				
Acute Myelogenous Leukemia	LeukoStrat CDx FLT3 Mutation Assay	IDT mutations and TKD mutations D835 and I836 in FLT3	Rydapt (midostaurin). Xospata (gilterinib). Vanflyta (quizartinib)	Konopleva et al. (2022)
Caris Life Sciences				
Colorectal Cancer	MI Cancer Seek	KRAS wild-type biomarkers (the absence of mutations in exons 2, 3, or 4) and NRAS wild-type biomarkers (the absence of mutations in exons 2, 3, or 4)	Vectibix (panitumumab)	Domenyuk et al. (2025)
		BRAF V600E	Braftovi (encorafenib) in combination with Erbitux (cetuximab)	Domenyuk et al. (2025)
Melanoma		BRAF V600E and V600K	Mekinist (trametinib)	Domenyuk et al. (2025)
Breast Cancer		PIK3CA variants including C420R, E542K, E545A, E545D, E545G, E545K, Q546E, Q546R, H1047L, H1047R, and H1047Y	Piqray (alpelisib)	Domenyuk et al. (2025)
Endometrial Carcinoma		Not Microsatellite instability-high (Not MSI-H)	Keytruda (pembrolizumab) in combination with Lenvima (lenvatinib)	Domenyuk et al. (2025)
Solid Tumors		Microsatellite instability – High (MSI-H)	Keytruda (pembrolizumab). Jemperli (dostarlimab-gxly)	Domenyuk et al. (2025)
MolecularMD Corporation				
Chronic Myeloid Leukemia	MRDx BCR-ABL Test	BCR-ABL fusion	Tasigna (nilotinib)	Toplin et al. (2013)
Pillar Biosciences, Inc.				
Colorectal Cancer	O/RDx-LCCA	KRAS wild type (absence of mutations in codons 12 and 13)	Erbitux (cetuximab). Vectibix (panitumumab)	Johnson et al. (2022)
NSCLC	​	Exon 19 deletion or exon 21 L858R substitution mutation	EGFR TKIs	Johnson et al. (2022)
Promega Corporation				
Endometrial Carcinoma	OncoMate MSI Dx Analysis System	Microsatellite stable/MSS (Not MSI-High)	Keytruda (pembrolizumab) in combination with Lenvima (lenvatinib)	Mendiola et al. (2025)

EGFR targeted therapy represents a key example of precision oncology enabled by CDx testing. EGFR is a transmembrane receptor tyrosine kinase involved in regulating cellular proliferation, survival, and differentiation. Activating mutations in the EGFR gene lead to constitutive receptor signaling and uncontrolled tumor growth particularly in non-small cell lung cancer (NSCLC). Clinically actionable EGFR alterations include the common activating mutations exon 19 deletions and the L858R substitution in exon 21, as well as less frequent variants such as exon 20 insertions, G719X, S768I, and L861Q (Fabrizio et al., 2024; Trinh and Abughanimeh, 2024). Identification of these genomic alterations through validated CDx assays are essential for selecting patients that are most likely to benefit from EGFR-targeted therapies. Small-molecule tyrosine kinase inhibitors (TKIs), including first (e.g., gefitinib), second (e.g., Afatinib), and third-generation agents (e.g., osimertinib) inhibit the intracellular kinase domain of EGFR and effectively suppress downstream oncogenic signaling in tumors harboring sensitizing EGFR mutations (Shaban et al., 2023; Araki et al., 2023). In contrast, monoclonal antibodies such as cetuximab target the extracellular domain of the receptor preventing ligand binding and receptor activation (Brand et al., 2011). The clinical efficacy of these agents is highly dependent on the presence of specific EGFR driver mutations underscoring the critical role of CDx-guided molecular testing in treatment selection. Increasing adoption of comprehensive genomic profiling has further expanded the clinical utility of CDx-guided therapeutic strategies in NSCLC. In early-stage (stage I–IIIA) NSCLC, molecular characterization enables the identification of actionable genomic alterations and facilitates the integration of targeted therapies in neoadjuvant, adjuvant, and perioperative treatment settings (Grant and Nagasaka, 2024). Biomarker-driven treatment approaches have demonstrated significant clinical benefit in multiple trials leading to regulatory approvals of targeted agents in early stages. Despite these advances, a substantial proportion of patients with newly diagnosed NSCLC still do not undergo comprehensive molecular testing or are screened for only a limited number of genomic alterations. This underutilization of CDx and genomic profiling may result in missed opportunities for biomarker-guided therapy and suboptimal clinical outcomes. Consequently, the integration of robust CDx platforms and comprehensive genomic profiling into routine clinical practice is essential to ensure that patients are appropriately matched to the most effective targeted therapies and clinical trials.

Rearrangements involving the ALK gene represent an important oncogenic driver in a subset of cancers, particularly NSCLC (Della Corte et al., 2018). These rearrangements typically result in fusion between EML4-ALK that produces a constitutively active fusion protein leading to persistent activation of downstream signaling pathways that promote uncontrolled cellular proliferation and survival (Lei et al., 2022). The identification of ALK rearrangements through validated CDx assays is therefore critical for selecting patients who are most likely to benefit from ALK-targeted therapies. The development of ALK TKIs has significantly transformed the treatment landscape for ALK-positive NSCLC. The first clinically approved ALK inhibitor crizotinib demonstrated superior efficacy compared with conventional chemotherapy in patients with ALK-rearranged advanced NSCLC (Cruz et al., 2021). Subsequent generations of ALK inhibitors including alectinib, brigatinib, and lorlatinib were developed to improve potency, overcome resistance mutations, and enhance brain penetration (Yang et al., 2020). Importantly, the clinical success of these targeted agents is highly dependent on accurate molecular identification of ALK rearrangements using CDx platforms such as fluorescence *in situ* hybridization, immunohistochemistry or NGS (Uruga and Mino-Kenudson, 2018; Letovanec et al., 2018). These diagnostic approaches enable precise patient stratification and ensure that individuals harboring ALK fusions are appropriately matched to effective targeted therapies exemplifying the central role of CDx in guiding precision oncology treatment strategies.

Mutations in the kirsten rat sarcoma viral oncogene homolog (KRAS) oncogene represent one of the most common oncogenic drivers in human malignancies and constitute the most frequently altered member of the RAS gene family. KRAS mutations occur across multiple tumor types with particularly high prevalence in NSCLC, pancreatic ductal adenocarcinoma, and colorectal cancer (Fois et al., 2021; Palomba et al., 2016; Luo, 2021). Importantly, the frequency of KRAS mutations vary substantially across cancer types reflecting the molecular heterogeneity of KRAS-driven tumors. This diversity has significant therapeutic implications and underscores the importance of precise molecular characterization through CDx assays to guide targeted treatment strategies. Historically, KRAS was considered “undruggable” due to its high affinity for GTP/GDP and the absence of suitable binding pockets for small-molecule inhibitors (Cox et al., 2014; Huang et al., 2021). As a result, early therapeutic efforts focused on targeting downstream effectors within the RAS signaling cascade, including the MAPK signaling pathway components such as RAF, MEK, and ERK (Ma et al., 2025). However, the marked biological heterogeneity of KRAS-mutant tumors limited the effectiveness of these indirect strategies. Advances in structural biology and drug discovery have recently enabled the development of mutation-specific inhibitors that selectively target distinct KRAS variants highlighting the growing importance of genotype-directed therapy within the precision oncology framework. The emergence of KRAS-targeted therapies has reinforced the critical role of CDx-guided molecular testing in therapeutic decision-making. Identification of specific KRAS mutations through validated diagnostic platforms allow clinicians to stratify patients for mutation-selective inhibitors and enrollment in biomarker-driven clinical trials. In addition to approved KRAS-targeted therapies, several next-generation inhibitors are currently under clinical investigation including daraxonrasib (RMC-6236), ADT-007, BI-3706674, JAB-23425, YL-1723, BI-2865, and BI-2493 (Jung et al., 2025; Foote et al., 2024; Bröker et al., 2025; Krupa et al., 2025; Tedeschi et al., 2025; Yang et al., 2024). These emerging agents aim to target a broader range of KRAS mutations or pan-RAS signaling further expanding the potential for CDx-enabled precision therapies.

Alterations in the BRAF gene represent an important oncogenic driver across several malignancies. BRAF encodes a serine/threonine kinase that functions within the MAPK/ERK signaling pathway (Poulikakos et al., 2022). Activating mutations in BRAF result in constitutive kinase activity and persistent downstream signaling leading to uncontrolled cell growth and tumorigenesis. Among these alterations, the BRAF V600E substitution is the most prevalent and well-characterized mutation accounting for the majority of BRAF-driven cancers (Bharti et al., 2025). Overall, BRAF mutations are estimated to occur in approximately 4%–8% of all malignancies with particularly high prevalence in melanoma and colorectal cancer. The detection of BRAF V600 mutations through validated CDx assays is essential for identifying patients who may benefit from BRAF-targeted therapies. Molecular testing platforms including PCR-based assays and NGS enable accurate identification of actionable BRAF alterations and guide therapeutic decision-making in clinical practice. Targeted inhibitors such as vemurafenib, dabrafenib, and encorafenib have received regulatory approval for the treatment of patients with advanced melanoma harboring BRAF V600 mutations (Proietti et al., 2020a; Eroglu and Ribas, 2016). Preclinical and clinical studies have demonstrated that these agents selectively inhibit mutant BRAF kinase activity resulting in suppression of ERK phosphorylation, inhibition of tumor cell proliferation, induction of G1 cell-cycle arrest, and promotion of apoptosis in BRAF-mutant melanoma cells (Proietti et al., 2020a; Eroglu and Ribas, 2016). Apart from the aforementioned well-known mutations identified through CDx assays, several other genetic variants identified through NGS are actionable. CDx assays have shown to identify genetic variants such as actionable mutations in genes such as RET, MET, CHEK2, BRCA, ERBB, FGFR, PIK3CA, PDGFRA and so on. Other than point mutations, CDx assays can identify gene fusions in RET, MET, ROS and amplification of genes such as EGFR, ERBB, CDK 4/6, AR and others (Table 4).

Despite remarkable advances in genomics-guided precision oncology and CDx assays, several biological and clinical limitations continue to restrict the long-term efficacy of targeted cancer therapies. Although genomic profiling has enabled the identification of actionable oncogenic mutations, durable responses remain limited in many patients due to intrinsic resistance, acquired resistance, tumor heterogeneity, concurrent mutations and adaptive evolutionary mechanisms (Li et al., 2025). One of the most well-characterized examples of acquired resistance is observed in EGFR mutant NSCLC. While first- and second-generation EGFR tyrosine kinase inhibitors TKIs initially demonstrate substantial clinical benefit, many patients eventually develop secondary resistance mutations such as T790M and C797S (Chhouri et al., 2023). These acquired mutations restore downstream signaling and disrupt therapeutic efficacy. Similarly, in BRAF V600E mutated melanoma, resistance to BRAF inhibitors frequently arises through reactivation of the MAPK pathway via secondary mutations in NRAS, MEK, or BRAF amplification ultimately resulting in disease progression (Proietti et al., 2020b). Adaptive bypass signaling through parallel pathways such as PI3K/AKT/mTOR further contributes to therapeutic failure and tumor survival. Concurrent mutations in STK11, KEAP1, TP53 along with oncogenic driver mutations or co-existing genetic alterations such as activation of bypass signaling pathways like MET amplification, EMT, and an immunosuppressive TME can lead to therapy resistance (Sumii et al., 2023). These findings highlight the complexity of resistance mechanisms in cancer and emphasize the importance of understanding the molecular pathways involved in tumor progression and therapeutic failure.

The clinical efficacy of genomics-guided therapies is also complicated by extensive intratumoral heterogeneity and clonal evolution. Tumors often consist of multiple genetically distinct subclones allowing resistant populations to emerge under therapeutic pressure (Zhang and Wang, 2025). Single-site biopsies may therefore fail to fully capture the molecular complexity of advanced malignancies by limiting the predictive accuracy of genomic profiling. Furthermore, actionable mutations do not always translate into clinical benefit emphasizing that genomic alterations alone may be insufficient predictors of therapeutic response. Several large precision oncology trials have highlighted these limitations. For example, the SHIVA trial demonstrated that molecularly targeted therapies selected solely on the basis of tumor molecular profiling did not significantly improve progression-free survival compared with physician’s choice of therapy across diverse advanced cancers (Le Tourneau et al., 2015). Similarly, umbrella and basket trials such as NCI-MATCH revealed that only a subset of genomically matched patients achieved meaningful clinical responses underscoring the complexity of translating genomic findings into effective therapies (O’Dwyer et al., 2023). Although genomics-driven precision oncology has revolutionized cancer treatment, substantial challenges remain in overcoming resistance mechanisms and improving the durability of therapeutic responses. Integrating longitudinal genomic monitoring, liquid biopsy, single-cell sequencing, spatial transcriptomics, and AI-based predictive modeling may help address these limitations and improve patient stratification and treatment optimization.

### 5.2 Immunogenomics

Immunogenomics is the study of genetic influence on the behavior of immune cells in both health and disease. It decodes the genetic and molecular signals that guide immune function with the goal of enabling novel diagnostics, therapies, and personalized treatment approaches (Liu and Mardis, 2017). Advances in high-throughput sequencing technologies have enabled comprehensive characterization of tumor genomes and the immune microenvironment providing critical insights into mechanisms of immune recognition, tumor immune evasion, and therapeutic response (Zheng and Zhang, 2021). In recent years, immunotherapy has emerged as a transformative approach in oncology aiming to restore or enhance the immune system’s ability to recognize and eliminate malignant cells (Garg et al., 2024). However, clinical responses remain heterogeneous with only a subset of patients deriving durable benefit (Wang, 2020). Immunogenomic analyses have been instrumental in elucidating the genetic and molecular features that influence therapeutic outcomes including alterations within the tumor microenvironment, immune signaling pathways, and antigen presentation (Li et al., 2020). For example, tumors with high mutational burdens often generate large numbers of tumor-specific neoantigens that increase tumor immunogenicity and enhance responsiveness to immune checkpoint blockade therapies (Sun et al., 2025).

Immune checkpoint inhibitors targeting programmed cell death protein 1 (PD-1), programmed death-ligand 1 (PD-L1), and cytotoxic T-lymphocyte associated protein 4 (CTLA-4) have revolutionized the treatment of multiple malignancies (Shiravand et al., 2022). Despite these advances, only a fraction of patients responds to immune checkpoint blockade underscoring the need for robust genomic and immunological biomarkers that can guide patient selection and therapeutic decision-making. Within the TME, specific immune cell subsets including CD4^+^ and CD8^+^ memory T cells and their specific gene signatures have been associated with improved responses to anti-CTLA-4 therapy (Damei et al., 2025). In contrast, natural killer cell populations have been linked to responsiveness to anti-PD-1–based therapies (Shaver et al., 2021). These findings highlight the importance of immunogenomics through integrating genomic, transcriptomic, and immune profiling to better understand determinants of treatment efficacy. Moreover, genomic alterations that disrupt immune signaling pathways can contribute to immune escape and resistance to immunotherapy. For example, interferon gamma can activate tumor escape mechanisms such as the upregulation of PD-L1 on the tumor cell thereby suppressing antitumor immune responses (Liu, 2025). Integrative immunogenomic analyses are therefore essential for identifying mechanisms of resistance and for designing next-generation immunotherapeutic strategies. Recent technological advances such as CRISPR-Cas9 gene editing–based functional genomic screens have further accelerated the discovery of genes involved in immune evasion and immunotherapy resistance (Djajawi et al., 2024). These unbiased genome-wide approaches enable systematic identification of molecular targets that may enhance immunotherapy responsiveness.

A major breakthrough by cancer genomics is the identification of tumor-specific neoantigens (Zhang Z. et al., 2021). Peptides derived from somatic mutations that are presented on the tumor cell surface by human leukocyte antigen (HLA) molecules represent neoantigens (Bassani-Sternberg et al., 2017). Because these neoantigens are absent in normal tissues, they represent highly specific targets for immune recognition and play a critical role in initiating tumor-specific immune responses. Advances in NGS, including WES and WGS allow the identification of patient-specific non-synonymous mutations that may generate neoantigens (Zhang Y. et al., 2025). Computational algorithms are then used to predict binding of mutated peptides to HLA molecules with sufficient affinity that can trigger T-cell responses. Although these predictive approaches have improved significantly, identifying truly immunogenic neoantigens remains challenging due to the complexity of antigen processing and presentation. Despite these challenges, neoantigen-based therapeutic strategies are rapidly emerging. Personalized neoantigen vaccines, synthetic peptide vaccines, and RNA-based vaccine platforms have demonstrated promising immunogenicity and clinical activity particularly in melanoma (Lang et al., 2022; Nguyen et al., 2025; Sahin et al., 2017). Additionally, neoantigen-specific T-cell receptors can be engineered *ex vivo* and reintroduced into patients through adoptive T-cell transfer enabling highly personalized cancer immunotherapy known as chimeric antigen receptor T-cell (CAR-T) therapy (Zhu et al., 2021).

Tumor mutational burden (TMB) has emerged as a clinically relevant collective genomic biomarker for predicting response to immunotherapy across multiple solid tumors (Sha et al., 2020). TMB refers to the total number of somatic mutations present within the coding regions of a tumor genome typically expressed as mutations per megabase of DNA. Tumors with elevated TMB are more likely to generate immunogenic neoantigens thereby increasing the probability of effective immune recognition and response to immune checkpoint inhibitors (Budczies et al., 2019). NGS has enabled more accurate and scalable measurement of TMB using targeted genomic panels and liquid biopsy approaches. Commercially available assays such as FoundationOne CDx and MSK-IMPACT allow comprehensive genomic profiling of tumors while simultaneously estimating TMB (Büttner et al., 2019). These platforms have facilitated the integration of genomic biomarkers into routine clinical practice and have supported regulatory approvals for immunotherapy in biomarker-selected patient populations. Nevertheless, several challenges remain including variability in TMB thresholds across tumor types, tumor heterogeneity, and the distinction between immunogenic and non-functional mutations. Ongoing research aims to refine composite biomarkers that integrate TMB with other genomic and immunological parameters such as neoantigen load and immune microenvironment signatures to improve prediction of immunotherapy response.

### 5.3 RNA-based therapeutics

Advances in cancer genomics have enabled the development of RNA-based therapeutic strategies that directly modulate gene expression at the post-transcriptional level. Among these approaches, small interfering RNA (siRNA)-based therapeutics have emerged as a promising modality for selectively silencing oncogenic drivers identified through genomic profiling (Ebenezer et al., 2025). Unlike conventional chemotherapeutic agents, RNA-based therapies offer high target specificity and the ability to inhibit disease-associated genes with potentially lower systemic toxicity. Despite their therapeutic potential, the clinical application of siRNA molecules initially faced significant challenges related to instability, rapid degradation in circulation, and inefficient intracellular delivery. To overcome these barriers, extensive efforts have been directed toward the development of chemical modifications and advanced delivery platforms. Lipid nanoparticle (LNP)-based delivery systems have proven particularly effective in protecting siRNA molecules from nuclease degradation and facilitating cytoplasmic delivery into target cells (El Moukhtari et al., 2023; Mainini and Eccles, 2020). These LNPs demonstrate high encapsulation efficiency, improved pharmacokinetics, and favorable biocompatibility compared with earlier nanoparticle formulations. Additional delivery strategies have also been developed to enhance tissue-specific targeting. For example, conjugation with triantennary N-acetylgalactosamine (GalNAc) ligands enables selective uptake by hepatocytes thereby increasing suitability for liver-directed therapies (Springer and Dowdy, 2018). In contrast, lipid nanoparticle systems allow broader delivery across multiple tissue types and are being explored for diverse cancer indications. Ongoing research is also investigating oral delivery systems and novel biomaterial carriers to improve patient compliance and overcome the limitations associated with injectable formulations (Ebenezer et al., 2025). While there is no cancer-specific siRNA that is approved by FDA, a few trials have investigated siRNA based therapeutics. siG12D-LODER™, a siRNA drug specific to KRAS G12D variant was investigated in an open-label Phase 1 study in non-operable locally advanced pancreatic cancer patients. Combination of siG12D-LODER™ with chemotherapy was shown to be well tolerated, safe and demonstrated a potential efficacy in patients (Golan et al., 2015). TKM-080301, siRNA targeting polo-like kinase 1 was investigated in an open-label, multicenter, phase I study involving advanced hepatocellular carcinoma patients (El et al., 2019). Though the study did not demonstrate significant anti-tumor efficacy but established the tolerability of siRNA based therapeutics in human patients.

Another important class of RNA-based therapeutics enabled by genomic discoveries is antisense oligonucleotides (ASOs). ASOs are short, synthetic single-stranded nucleic acid molecules designed to bind complementary RNA sequences and modulate gene expression (Collotta et al., 2023). Their high specificity arises from the ability to selectively target transcripts encoded by disease-associated genes identified through genomic sequencing. The clinical potential of antisense technologies has been recognized for several decades. In 1998, the first ASO, fomivirsen, received approval from the US-FDA for the treatment of cytomegalovirus retinitis in patients with AIDS (Ding et al., 2025). This breakthrough established the therapeutic feasibility of oligonucleotide-based medicines. Since then, numerous antisense drugs have been approved for genetic and metabolic disorders. ASO based therapeutics expanded rapidly with the development of second and third generation chemically modified ASOs that exhibit improved stability, enhanced target affinity, and reduced immunogenicity (Ding et al., 2025). While ASOs have gained FDA approval for rare genetic, neurological, and metabolic diseases, their application in oncology remains primarily in clinical trials (Xiong et al., 2021). Many ASOs are in phase 1 or 2 trials, with some in phase 3 and advanced trials such as oblimersen, PNT2258, cobomarsen, BP1001, and IMV-001 (Walker et al., 2021; Harb et al., 2021; Anastasiadou et al., 2021; Ohanian et al., 2018; Lee et al., 2024). The therapeutic targeting of miRNAs represents a major translational milestone. A phase 1 trial evaluated the safety and efficacy of MRX34, a liposomal miRNA-based cancer therapy mimicking miRNA-34a in patients with advanced solid tumors (Hong et al., 2020). Trial provided proof-of-concept for miRNA-based dose dependent modulation of relevant target genes. miRNA therapeutics in cancer are currently in early-phase (Phase I/II) clinical trials focusing on restoring tumor-suppressive miRNAs (mimics) or inhibiting oncogenic miRNAs (antimiRs). Key miRNA based therapeutics in ongoing clinical trials are MRX34, MesomiR-1 (miR-16 mimic), TTX-MC138, and miR-193a-3p mimic (Reid et al., 2016; Halim et al., 2025; Telford et al., 2021). Collectively, these technological advances illustrate how genomic knowledge of cancer-associated genes can be translated into highly specific RNAi-based therapeutic strategies.

### 5.4 Cancer vaccines

Recent advances in cancer genomics, NGS, and immunogenomics in neoantigen prediction have substantially accelerated the development of personalized vaccine platforms. Cancer vaccines have emerged as a promising therapeutic strategy in precision oncology by harnessing the immune system to generate durable and tumor-specific antitumor responses. Unlike prophylactic vaccines that prevent virus-associated malignancies, therapeutic cancer vaccines are designed to stimulate immune responses against existing tumors through the presentation of tumor-associated antigens or tumor-specific neoantigens (Rota et al., 2026). Despite earlier limitations associated with immune tolerance, tumor heterogeneity, antigenic variability, and insufficient immunogenicity, recent innovations in nucleic acid delivery systems, adjuvant technologies, and combination immunotherapies have renewed interest in cancer vaccine development (Huang et al., 2026). Furthermore, combining cancer vaccines with immune checkpoint inhibitors, radiotherapy, or targeted therapies has demonstrated synergistic effects by enhancing antigen presentation, reversing immune suppression within the TME, and promoting durable antitumor immunity. Radiotherapy, in particular, has been shown to enhance tumor immunogenicity through immunogenic cell death and increased neoantigen release thereby potentiating vaccine-induced immune responses (Lhuillier et al., 2021). Cancer vaccines are categorized into two main groups: preventive (prophylactic) vaccines and therapeutic vaccines. A shift in focus on therapeutic vaccines in the recent past is witnessed due to its customizable ability. More studies so far including the real-world human patient clinical trials have been on cell-based, peptide-based and nucleic-acid based (DNA/RNA vaccines) therapeutic vaccines.

#### 5.4.1 DNA-based vaccines

DNA-based cancer vaccines utilize genetically engineered plasmid DNA encoding tumor-associated antigens to induce antigen-specific immune responses. Following cellular uptake, the plasmid enters the nucleus where transcription of the encoded antigen occurs leading to antigen presentation through major histocompatibility complex (MHC) pathways. However, efficient intracellular delivery remains a major challenge because plasmid DNA must overcome multiple biological barriers including cellular uptake, endosomal escape, and nuclear membrane transport (Moseman et al., 2025). Recent advances in vector engineering have led to the development of next-generation DNA vaccine platforms such as doggybone DNA, minicircle DNA, and nanoplasmid DNA which demonstrate improved transgene expression, reduced bacterial sequences, and enhanced safety profiles compared with conventional plasmid systems (Wu A. A. et al., 2025).

#### 5.4.2 RNA-based vaccines

Among emerging vaccine platforms, mRNA-based vaccines have gained considerable attention owing to their favorable safety profile, rapid manufacturing capability, and strong immunogenic potential. mRNA vaccines encode tumor-specific antigens or neoantigens and enable transient antigen expression without the risk of genomic integration (Malla et al., 2024). In addition, mRNA molecules possess intrinsic adjuvant activity through activation of innate immune receptors such as Toll-like and RIG-I–like receptors leading to robust activation of dendritic cells and antigen-presenting pathways (Verbeke et al., 2022). These vaccines can induce potent CD8 and CD4 T-cell responses that are critical for durable antitumor immunity. Recent advances in LNP delivery systems and self-amplifying RNA technologies have further improved the stability, translational efficiency, and immunogenicity of RNA vaccines. Personalized neoantigen mRNA vaccines are currently being evaluated in multiple clinical trials, particularly in melanoma, pancreatic cancer, and NSCLC (Li X. et al., 2026). Importantly, combination strategies involving mRNA vaccines and immune checkpoint blockade have demonstrated promising clinical activity and prolonged recurrence-free survival in melanoma and NSCLC patients highlighting the translational potential of genomics-guided personalized vaccine therapies (Li X. et al., 2026).

#### 5.4.3 Peptide-based vaccines

Peptide-based cancer vaccines represent one of the earliest forms of therapeutic cancer vaccination and are designed to stimulate T cell responses against defined tumor-associated peptide epitopes. However, their clinical efficacy has historically been limited by low immunogenicity, rapid peptide degradation, and MHC restriction. Peptide presentation depends on specific HLA haplotypes (Andersen, 2026). Genetic variability in HLA determines the successful or failed presentation of peptide antigen that leads to limited reliability on peptide vaccines. To overcome these limitations, recent strategies have focused on utilizing HLA supertypes, multi-epitope formulations, long synthetic peptides, and potent adjuvants to broaden immune coverage and improve immunogenicity. Several peptide-based vaccines targeting antigens such as survivin, telomerase, HER2, and WT1 are currently under clinical evaluation (Andersen, 2026). In addition, neoantigen-based peptide vaccines derived from patient-specific genomic profiling have shown considerable promise in inducing personalized antitumor T-cell responses. Advances in AI-driven epitope prediction and immunopeptidomics are expected to further refine peptide vaccine design and improve therapeutic efficacy.

#### 5.4.4 Cell-based vaccines

Tumor cells are a prominent source of antigens for cancer vaccine preparations. They can be used either as whole tumor cells or through the generation of tumor lysates. One of the most effective forms of using tumor cells for preparation of cancer vaccines is through the use of dendritic cell-based vaccines (Santos and Butterfield, 2018). Due to their potent antigen-presenting capacity and ability to activate naïve T cells, dendritic cell-based vaccines have demonstrated the clinical success (Santos and Butterfield, 2018). In clinical settings, dendritic cell-based vaccines are typically generated from peripheral blood monocytes or hematopoietic progenitor cells cultured with cytokines to induce differentiation into mature dendritic cells (Santos and Butterfield, 2018). These cells are subsequently loaded with tumor antigens, peptides, tumor lysates, or nucleic acids before reinfusion into patients. A major milestone in cancer vaccine therapy was the FDA approval of Sipuleucel-T for metastatic castration-resistant prostate cancer (Kantoff et al., 2010). Sipuleucel-T utilizes autologous antigen-presenting cells activated with the recombinant fusion protein PA2024 and demonstrated improved overall survival in patients with advanced prostate cancer. Other cell-based vaccine approaches under investigation include natural killer cell-based vaccines, B-cell vaccines, and genetically modified whole tumor cell vaccines. Natural killer cell-based strategies have gained increasing attention due to their intrinsic antitumor activity and ability to reshape the tumor microenvironment toward a pro-inflammatory state particularly when combined with Toll-like receptor activation (Toffoli et al., 2023). Several investigational vaccines have also shown encouraging clinical outcomes. For example, gemogenovatucel-T, a genetically modified autologous tumor cell vaccine targeting furin and TGF-β1/2 demonstrated reduced recurrence risk in patients with advanced ovarian cancer (Rocconi et al., 2020).

#### 5.4.5 *In situ* cancer vaccines

*In situ* cancer vaccination strategy utilizes the tumor itself as a source of endogenous antigens to induce systemic antitumor immunity. This approach relies on localized therapies such as radiotherapy, photothermal therapy, chemotherapy, oncolytic viruses, or intratumoral immunostimulatory agents to induce immunogenic tumor cell death and enhance antigen presentation within the TME (Giram et al., 2025). The subsequent activation of innate and adaptive immune responses transforms the tumor into an endogenous vaccine depot capable of generating systemic immune surveillance. A notable example of an FDA-approved *in situ* vaccine strategy is Talimogene Laherparepvec, an engineered oncolytic herpes simplex virus approved for the treatment of advanced melanoma (Ferrucci et al., 2021). Talimogene Laherparepvec engineered to preferentially infect and destroy cancer cells while stimulating a systemic anti-tumor immune response (Ferrucci et al., 2021).

### 5.5 CRISPR-based therapeutics

The discovery of the CRISPR-Cas9 genome editing system has revolutionized the field of cancer biology and precision medicine by enabling targeted modification of disease-associated genes. Originally identified as an adaptive immune defense mechanism in bacteria and archaea, CRISPR technology enables precise genome editing through a gRNA that directs the Cas nuclease to a specific DNA sequence resulting in a double-strand break and subsequent DNA repair via non-homologous end joining or homology-directed repair. These mechanisms allow the disruption of oncogenes, correction of pathogenic mutations, or insertion of therapeutic sequences within the genome (Ding et al., 2023; Chehelgerdi et al., 2024). In cancer research, CRISPR-based genome editing has become a powerful tool for investigating tumor biology and identifying genetic vulnerabilities. The technology allows systematic interrogation of oncogenes, tumor suppressor genes, and regulatory elements that contribute to tumorigenesis. Genome-wide CRISPR screening approaches have facilitated the discovery of novel therapeutic targets and resistance mechanisms to existing therapies. As a result, CRISPR is increasingly viewed not only as a research tool but also as a promising therapeutic strategy capable of directly targeting the genetic drivers of cancer (Sharma and Giri, 2024).

CRISPR-based therapeutics in oncology can be broadly categorized into three main strategies: direct gene editing of tumor cells, engineering of immune cells for cancer immunotherapy, and functional genomic screening to identify novel therapeutic targets. One strategy involves the direct disruption of oncogenic mutations that drive tumor growth. For example, CRISPR systems have been used experimentally to inactivate oncogenes such as KRAS, MYC, and EGFR, or restore tumor suppressor genes such as TP53. Preclinical studies have demonstrated that CRISPR-mediated editing of these genes can suppress tumor proliferation and induce apoptosis in cancer cells (Rehman and Abbas, 2026). Another major application involves engineering immune cells to enhance antitumor immunity. CRISPR has been widely used to modify T cells by deleting immune checkpoint genes such as PD-1 or other regulatory molecules that limit T-cell activity. Genome-edited T cells exhibit improved persistence, enhanced tumor recognition, and stronger cytotoxic responses against malignant cells. These approaches have contributed to the development of next-generation cell therapies such as CRISPR-engineered CAR-T cells and tumor-infiltrating lymphocytes (TILs) (Kavousinia et al., 2024). Additionally, CRISPR-based functional genomic screens have enabled the identification of genes that regulate tumor immune evasion, drug resistance, and metastatic potential. These studies have significantly expanded the repertoire of potential therapeutic targets and have accelerated drug discovery efforts in precision oncology (Han et al., 2025).

In recent years, significant progress has been made in developing CRISPR-based therapeutic platforms for cancer treatment. Advances in gRNA design, delivery systems, and genome editing accuracy have improved the feasibility of translating CRISPR technologies into clinical applications. Various delivery strategies, including viral vectors, lipid nanoparticles, and electroporation-based *ex vivo* editing approaches are currently being explored to efficiently deliver CRISPR components into target cells. One promising area of development is CRISPR-based immunotherapy. Gene editing technologies are being used to enhance T-cell function by knocking out inhibitory signaling pathways or introducing tumor-specific receptors. CRISPR-engineered immune cells have demonstrated improved antitumor activity in several preclinical models, highlighting the potential of genome editing to overcome tumor-induced immune suppression (Feng et al., 2024). In addition, next-generation CRISPR systems such as base editors and prime editing technologies are being investigated for their ability to introduce precise nucleotide changes without generating double-strand DNA breaks (Zeballos and Gaj, 2021). These innovations may reduce off-target effects and improve the safety of genome editing therapies in clinical settings.

The translation of CRISPR technology into clinical oncology has progressed rapidly over the past decade. Several early-phase clinical trials have evaluated the safety and feasibility of CRISPR-edited immune cells for cancer treatment. One of the earliest CRISPR clinical trials involved editing T cells to disrupt the immune checkpoint gene PD-1 in patients with advanced cancers (Lu et al., 2020). The edited T cells demonstrated feasibility and acceptable safety profiles in early clinical studies. Similar strategies have been explored in CRISPR-engineered CAR-T cells targeting hematological malignancies such as leukemia and lymphoma (Tao et al., 2024). Recently, a first-in-human clinical trial evaluated CRISPR-edited TILs in patients with metastatic gastrointestinal cancers. In this study, CRISPR/Cas9 was used to disrupt the intracellular immune checkpoint gene CISH in patient-derived TILs, enhancing their ability to recognize and eliminate tumor cells (Lou et al., 2025). The modified T cells were expanded *ex vivo* and reinfused into patients. Trial demonstrated encouraging safety results including disease stabilization in several patients and a complete response in one patient. Several additional CRISPR-based immunotherapy trials are currently underway exploring genome editing strategies to enhance T-cell persistence, reduce immune exhaustion, and improve tumor targeting. These studies represent important steps toward establishing CRISPR-based cell therapies as a feasible component of precision oncology.

### 5.6 CAR T-cell therapy

CAR-T cell therapy represents a transformative advancement in precision immuno-oncology in which a patient’s autologous T lymphocytes are genetically engineered to recognize and eliminate tumor cells expressing specific surface antigens. CAR-T cells are generated by isolating patient-derived T cells, genetically modifying them *ex vivo* to express synthetic chimeric antigen receptors (Brudno et al., 2024). Engineered T cells with CAR constructs are then reinfused back into the patient to mediate targeted cytotoxicity against tumor-associated antigens such as EGFR, mesothelin, HER2, GD2, PD-L1 and so on (Table 2). This personalized immunotherapeutic approach has demonstrated remarkable clinical success in hematological malignancies, particularly B-cell leukemias and lymphomas. However, the therapeutic efficacy of CAR-T therapy in solid tumors remains limited owing to challenges including antigen heterogeneity, poor tumor infiltration, immunosuppressive TME, T-cell exhaustion, antigen escape, and treatment-associated toxicities (Escobar et al., 2025). Recent advances in cancer genomics and genome engineering technologies particularly CRISPR-Cas9 gene editing and single-cell sequencing approaches are substantially improving CAR-T cell design, target identification, resistance profiling, and therapeutic safety. CRISPR-mediated genome editing now enables precise engineering of CAR-T cells through targeted disruption of inhibitory immune checkpoint molecules such as PD-1, elimination of endogenous T-cell receptor expression to generate allogeneic “off-the-shelf” CAR-T products (Dimitri et al., 2022). Furthermore, multiplex CRISPR editing strategies are being explored to simultaneously enhance persistence, reduce exhaustion, and improve resistance to immunosuppressive signaling within the TME (Murray et al., 2025).

A major limitation in solid tumor CAR-T therapy is insufficient trafficking and infiltration of engineered T cells into tumor tissues (Escobar et al., 2025). To overcome this barrier, several next-generation CAR-T engineering approaches are under investigation. For example, EGFR-targeted CAR-T cells co-expressing the chemokine receptor CXCR5 have demonstrated enhanced tumor homing and improved antitumor activity in preclinical solid tumor models (Li et al., 2021). Similarly, B7-H3 targeted CAR-T therapies have emerged as promising candidates for solid tumors because B7-H3 is highly expressed across multiple malignancies while exhibiting limited expression in normal tissues. A preclinical study demonstrated that B7-H3-DAP12 CAR-T cells significantly enhanced cytotoxicity against lung squamous cell carcinoma through DAP12-mediated activation signaling. Several ongoing clinical trials are currently evaluating the safety and efficacy of B7-H3–targeted CAR-T therapies in solid tumors (Table 1). Despite these advances, CAR-T cell therapy remains associated with several clinically significant toxicities. One of the most important limitations is on-target off-tumor toxicity which occurs when the targeted antigen is also expressed on normal tissues. A classic example is the depletion of normal B cells following CD19-directed CAR-T therapy resulting in prolonged B-cell aplasia (Kochenderfer and Rosenberg, 2013). Additionally, cross-reactivity against antigens expressed by healthy tissues may lead to unintended tissue injury. Other adverse effects associated with CAR-T infusion include tumor lysis syndrome, acute hypersensitivity reactions, and immune-mediated toxicities. Among these, cytokine release syndrome remains the most common and best characterized complication arising from excessive systemic release of inflammatory cytokines. CAR-T activation-induced cytokine release syndrome is characterized clinically by fever, hypotension, capillary leak, and systemic inflammatory responses (Brudno and Kochenderfer, 2016). Neurotoxicity, commonly referred to as immune effector cell associated neurotoxicity syndrome is another major complication of CAR-T therapy. Neurological manifestations can include encephalopathy, seizures, aphasia, cerebral edema, and altered mental status. These neurotoxic symptoms may occur independently of cytokine release syndrome suggesting distinct pathogenic mechanisms. The detection of CAR-T cells and elevated inflammatory cytokines such as IL-6 in cerebrospinal fluid indicates that the central nervous system immune activation contributes significantly to CAR-T-associated neurotoxicity (Brudno and Kochenderfer, 2016).

The integration of genomic technologies such as scRNA-seq, T- and B-cell receptor sequencing and CRISPR-based functional genomics has substantially accelerated the development of safer and more effective CAR-T therapies (Haradhvala and Maus, 2024). scRNA-seq enables high-resolution characterization of tumor heterogeneity, immune cell composition, antigen expression patterns, and exhaustion-associated transcriptional programs at the single-cell level (Haradhvala and Maus, 2024). These analyses facilitate identification of tumor-specific antigens while minimizing off-target toxicity by distinguishing malignant from healthy cellular populations. Furthermore, scRNA-seq combined with T- and B-cell receptor sequencing enables comprehensive profiling of immune repertoires, including clonal expansion, antigen specificity, T-cell activation states, exhaustion markers, and tumor-reactive lymphocyte populations within the TME (Haradhvala and Maus, 2024). These approaches are particularly valuable for identifying mechanisms of antigen escape and adaptive resistance that contribute to treatment failure. CRISPR-based pooled functional genomic screens are also being increasingly utilized to identify genes regulating CAR-T sensitivity, immune evasion, and T-cell dysfunction (Xiang et al., 2024). Such studies have identified multiple pathways associated with T-cell exhaustion, metabolic suppression, and resistance to cytotoxic lymphocyte activity thereby revealing novel targets for next-generation CAR-T engineering strategies (Xiang et al., 2024). In addition, emerging multimodal approaches integrating spatial transcriptomics, proteomics, and AI-assisted computational modeling are expected to further improve target prioritization, safety prediction, and precision immunotherapy design. Collectively, the combination of CRISPR genome engineering, single-cell genomics, and advanced immunoprofiling technologies is driving the development of next-generation CAR-T therapies with improved specificity, persistence, and efficacy against both haematological and solid malignancies. Although substantial challenges remain, continued advances in genomic technologies and synthetic immunology are expected to expand the therapeutic applicability of CAR-T cells and accelerate the evolution of precision immuno-oncology.

## 6 Limitations in genomics-guided precision oncology: real-world challenges

Despite remarkable advances in cancer genomics and precision oncology, the successful clinical implementation of genomics-guided cancer care remains constrained by substantial economic, regulatory, infrastructural, and operational barriers (Lajmi et al., 2024). Although NGS, CDx, and molecularly targeted therapies have transformed cancer management, their integration into routine clinical practice remains uneven across geographic regions and healthcare settings. Consequently, significant disparities persist in access to genomic testing, precision therapeutics, and advanced oncology infrastructure, particularly in low and middle income countries (Ruge et al., 2026). Major hurdles in successful implementation of genomic-guided practice into real-world include underutilization of genomic testing, cost and accessibility disparities, regulatory and companion diagnostic challenges, clinical adoption gaps, Infrastructure and expertise limitations.

### 6.1 Underutilization of genomic testing

NGS based molecular profiling has revolutionized oncology by enabling identification of actionable genomic alterations and personalized therapeutic strategies. However, genomic testing remains substantially underutilized in routine clinical practice despite its demonstrated clinical utility. Several interconnected factors contribute to this gap between technological capability and real-world implementation. Major barriers include inconsistent reimbursement policies, high out-of-pocket costs, administrative burdens associated with prior authorization processes, limited clinician awareness regarding genomic interpretation, and variability in institutional testing practices (Hussain et al., 2025). Furthermore, fragmented reporting systems, proprietary genomic databases, and lack of standardized bioinformatics pipelines complicate interpretation of genomic findings and reduce confidence in clinical decision making (Hussain et al., 2025). Collectively, these challenges create a translational bottleneck in which potentially actionable genomic information does not consistently translate into optimal treatment selection for patients. Additionally, disparities in genomic education among healthcare providers, limited access to molecular tumor boards, and insufficient integration of genomic data into electronic medical systems further hinder adoption of precision oncology. In many healthcare settings, clinicians may lack adequate training to interpret complex genomic reports or integrate molecular findings into therapeutic planning. These challenges are further amplified in resource-limited regions where specialized molecular pathology services and genomic counseling infrastructure remain underdeveloped (Hussain et al., 2025).

### 6.2 Cost and accessibility disparities

Economic inequality represents one of the most significant barriers to equitable implementation of precision oncology worldwide. The global cancer burden is projected to rise substantially over the coming decades with a substantial projected rise of newly diagnosed cancer cases in low and middle income countries by 2050 (Balch et al., 2026). However, advanced genomic diagnostics, targeted therapies, immunotherapies, and cell-based therapeutics remain disproportionately concentrated in high income countries. The Asia-Pacific region, which already accounts for a substantial proportion of global cancer-related mortality, continues to face major deficiencies in access to advanced radiotherapy, molecular diagnostics, and precision therapeutics (Martina and Segelov, 2023). Despite decrease in sequencing costs over the past decade, comprehensive genomic profiling, liquid biopsy assays, and personalized therapies such as CAR-T cells and neoantigen vaccines remain expensive for many healthcare systems. Emerging personalized immunotherapeutic strategies, including neoantigen targeting vaccines and CRISPR-engineered cell therapies require highly individualized manufacturing pipelines, sophisticated laboratory infrastructure, and complex quality control systems that substantially increase treatment costs. Consequently, the translational gap between technological innovation and socioeconomic feasibility remains a major challenge in global oncology. Bridging this gap will require coordinated investment in healthcare infrastructure, equitable reimbursement frameworks, international collaboration, and scalable manufacturing platforms capable of reducing the cost of personalized cancer therapies.

### 6.3 Regulatory and companion diagnostic challenges

The rapid expansion of genomics-guided therapeutics has also introduced significant regulatory complexity. Regulatory bodies such as the US-FDA, European Medicines Agency, and Pharmaceuticals and Medical Devices Agency have implemented regulatory pathways for precision therapeutics and companion diagnostics (Kadam et al., 2026). However, substantial variability persists across countries regarding approval timelines, reimbursement policies, and clinical implementation standards. As a result, access to novel targeted therapies remains highly uneven globally. A major challenge in precision oncology is the limited availability of analytically and clinically validated companion diagnostics. Delays in co-development of therapeutics and corresponding CDx assays often prolong the time required for patient stratification and targeted treatment initiation. In addition, regulatory frameworks frequently prioritize traditional clinical efficacy endpoints while underrepresenting patient-reported outcomes, quality of life metrics, and real-world evidence (Bellino and La Salvia, 2024). Increasing emphasis is now being placed on integrating patient-centric outcomes and standardized patient-reported outcomes into regulatory and health technology assessment pathways to facilitate more equitable and clinically meaningful implementation of precision therapies (Bellino and La Salvia, 2024). Another important limitation involves population diversity in genomic research and clinical trials. Many precision oncology studies continue to underrepresent ethnically diverse populations thereby raising concerns regarding generalizability of genomic biomarkers and therapeutic efficacy across global populations (Zhao et al., 2026). Failure to adequately account for ethnic and population specific genomic variability may contribute to disparities in treatment outcomes and limit the universal applicability of precision oncology approaches.

### 6.4 Clinical adoption gaps

Effective implementation of precision oncology requires not only genomic and technological innovations but also coordinated integration into patient care. Standard clinical workflows, multidisciplinary expertise, patient engagement, and healthcare policy support collectively contribute to the successful implementation of genomics-guided precision medicine. In many clinical settings, barriers such as insufficient personnel, limited consultation time, inadequate reimbursement models, and logistical constraints continue to restrict widespread adoption of genomics-guided care. Patient-reported outcomes and patient-centered oncology models are increasingly recognized as essential components of precision medicine because they improve symptom monitoring, treatment adherence, and quality of life assessment (Bellino and La Salvia, 2024). However, implementation of patient-reported outcomes driven clinical systems remains challenging due to time constraints, personnel shortages, data management limitations, and geographic disparities in healthcare access (Bellino and La Salvia, 2024). In low and middle income countries, additional barriers include limited biotechnology infrastructure, lack of population-scale genomic databases, shortages of trained molecular oncology specialists, and inadequate funding for translational research. These factors collectively impede the implementation of precision oncology programs and contribute to persistent global disparities in cancer outcomes.

### 6.5 Infrastructure and expertise limitations

The implementation of precision oncology is highly dependent on access to advanced laboratory infrastructure, high-throughput sequencing platforms, computational resources, and specialized multidisciplinary expertise. However, many healthcare systems, particularly in resource limited settings, lack the infrastructure required to support large-scale genomic testing, molecular pathology, bioinformatics analysis, and cell-based therapeutic manufacturing. Consequently, personalized cancer care remains inaccessible to large segments of the global population. The increasing complexity of genomic medicine has also increased the need for trained personnel capable of interpreting molecular data and integrating genomic findings into clinical management (Molla and Bitew, 2024). Molecular pathologists, clinical geneticists, bioinformaticians, computational biologists, and precision oncology specialists are globally scarce. Although digital oncology, teleoncology, and AI-assisted decision-support systems have emerged as potential solutions for addressing expertise shortages, integration of these technologies into routine clinical workflows remains challenging due to interoperability issues, data standardization concerns, and variability in data quality (Heudel et al., 2025).

Collectively, these challenges highlight that the future success of precision oncology will depend not only on continued technological innovation but also on equitable global implementation, standardized regulatory frameworks, sustainable healthcare investment, and development of robust clinical and computational infrastructure. Addressing these barriers will be essential to ensure that the benefits of cancer genomics and personalized therapeutics can be translated effectively into routine clinical care across diverse healthcare systems worldwide.

## 7 Conclusion and future perspectives

Advances in genomic technologies have fundamentally transformed the landscape of cancer research and clinical oncology. The integration of NGS, large-scale cancer genomics initiatives, and computational analysis has enabled the systematic identification of oncogenic drivers, resistance mechanisms, and clinically actionable biomarkers. These discoveries have facilitated the transition from conventional treatment paradigms toward genotype-guided precision oncology where therapeutic decisions are increasingly informed by the molecular characteristics of individual tumors rather than by histopathological classification alone. CDx platforms have played a pivotal role in translating genomic discoveries into clinical practice by enabling accurate identification of patients that are most likely to benefit from targeted and immunotherapies. In parallel, immunogenomic approaches have provided new insights into tumor-immune interactions and enabled the identification of biomarkers predicting response to immune checkpoint blockade. Similarly, RNA-based therapeutics and CRISPR-mediated genome editing technologies offer novel strategies for selectively targeting oncogenic pathways that were previously considered undruggable. Collectively, these developments highlight the important role of genomic technologies in driving innovation in cancer research, diagnosis, prognosis and treatment. The future of precision oncology will be continued integration of multi-omics technologies, advanced computational analytics, and innovative therapeutic platforms. Emerging approaches such as single-cell sequencing, spatial transcriptomics, and long-read sequencing are expected to provide unprecedented insights into tumor heterogeneity, clonal evolution, and microenvironmental interactions that influence disease progression and therapeutic response. The rapid progress in genome editing technologies also presents exciting opportunities for cancer therapy. Next-generation CRISPR systems including base editing and prime editing may enable precise correction or disruption of oncogenic mutations while minimizing off-target effects. Similarly, CRISPR-engineered immune cell therapies have the potential to enhance antitumor immunity and overcome mechanisms of immune escape. Finally, AI, ML, and DL-based data analytics platforms are expected to be an integral part of cancer genomics in future. Integration of AI-based and AI-driven analytical framework reduces data analysis time and human error. Together, these advances promise to further accelerate the transition toward highly personalized, genomics-guided cancer prevention, diagnosis, and treatment.

## Glossary

NGS: Next-Generation Sequencing
WGS: Whole-genome sequencing
WES: Whole-exome sequencing
US-FDA: United States Food and Drug Administration
TCGA: The Cancer Genome Atlas
EGFR: Epidermal Growth Factor Receptor
BRAF: B-type rapidly accelerated fibrosarcoma
MEK: Mitogen-activated extracellular signal-regulated kinase
ALK: Anaplastic lymphoma kinase
RNA-seq: RNA-sequencing
cDNA: Complementary DNA
ncRNAs: Noncoding RNAs
sncRNAs: Small non-coding RNAs
lncRNAs: Long non-coding RNAs
circRNAs: Circular RNAs
miRNAs: microRNAs
siRNAs: small interfering RNAs
piRNAs: PIWI-interacting RNAs
HOTAIR: Hox transcript antisense intergenic RNA
TME: Tumor microenvironment
scRNA-seq: Single-cell RNA sequencing
snRNA-seq: Single-nuclear RNA sequencing
CITE-seq: Cellular indexing of transcriptomes and epitopes by sequencing
ATAC-seq: Assay for transposase-accessible chromatin using sequencing
CDKN2A: Cyclin-dependent kinase inhibitor 2A
MLH1: MutL homolog 1
BRCA1: Breast cancer gene 1
ChIP-seq: Chromatin immunoprecipitation sequencing
HDACs: Histone deacetylases
EZH2: Enhancer of zeste homolog 2
KMT2D: Lysine methyltransferase 2D
ARID1A: AT-rich interaction domain 1A
ctDNA: Circulating tumor DNA
CTCs: Circulating tumor cells
cfDNA: Cell-free DNA
cfRNA: Cell-free RNA
MRD: Minimal residual disease
EVs: Extracellular vesicles
CRISPR: Clustered regularly interspaced palindromic repeats
gRNA: Guide RNA
AI: Artificial intelligence
ML: Machine learning
DL: Deep learning
PRS: Polygenic risk scores
SNPs: Single nucleotide polymorphisms
GWAS: Genome-wide association studies
MMR: Mismatch repair
HPV: Human papillomavirus
EBV: Epstein–Barr virus
SERMs: Selective estrogen receptor modulators
CDx: Companion diagnostic
NSCLC: Non-small cell lung cancer
TKIs: Tyrosine kinase inhibitors
KRAS: Kirsten rat sarcoma viral oncogene homolog
GTP: Guanosine triphosphate
GDP: Guanosine diphosphate
MAPK: Mitogen-activated Protein Kinase
ERK: Extracellular signal-regulated Kinase
RET: Rearranged during transfection
CHEK2: Checkpoint Kinase 2
ERBB: Erythroblastic oncogene B
FGFR: Fibroblast growth factor receptor
PIK3CA: Phosphatidylinositol-4,5-bisphosphate 3-kinase catalytic subunit-α
PDGFRA: Platelet-derived growth factor receptor-α
AR: Androgen receptor
PD-1: Programmed cell death protein 1
PD-L1: Programmed death-ligand 1
CTLA-4: Cytotoxic T-lymphocyte associated protein 4
HLA: Human leukocyte antigen
CAR-T: Chimeric antigen receptor T-cell
TMB: Tumor mutational burden
siRNA: small interfering RNA
LNP: Lipid nanoparticle
GalNAc: Triantennary N-acetylgalactosamine
ASOs: Antisense oligonucleotides
MYC: Myelocytomatosis viral oncogene homolog
TP53: Tumor protein 53
TILs: Tumor-infiltrating lymphocytes
CISH: Cytokine inducible SH2-containing protein
CHIEF: Clinical Histopathology Imaging Evaluation Foundation
EMT: Epithelial-mesenchymal transition
MHC: Major histocompatibility complex
